# Revision of *Gangesia* (Cestoda: Proteocephalidea) in the Indomalayan Region: Morphology, Molecules and Surface Ultrastructure

**DOI:** 10.1371/journal.pone.0046421

**Published:** 2012-10-03

**Authors:** Anirban Ash, Tomáš Scholz, Alain de Chambrier, Jan Brabec, Mikuláš Oros, Pradip Kumar Kar, Shivaji Prabhakar Chavan, Jean Mariaux

**Affiliations:** 1 Institute of Parasitology, Biology Centre of the Academy of Sciences of the Czech Republic & Faculty of Science, University of South Bohemia, České Budějovice, Czech Republic; 2 Department of Invertebrates, Natural History Museum, Geneva, Switzerland; 3 Institute of Parasitology, Slovak Academy of Sciences, Košice, Slovakia; 4 Jhargram Raj College, Jhargram, Paschim Medinipur, West Bengal, India; 5 School of Life Sciences, Swami Ramanand Teerth Marathwada University, Nanded, Maharashtra, India; Centro de Investigación y de Estudios Avanzados, Mexico

## Abstract

Tapeworms of *Gangesia* Woodland, 1924 (Cestoda: Proteocephalidea) parasitic in freshwater fishes in the Indomalayan Region were critically reviewed. Evaluation of type specimens and newly collected materials from Bangladesh, Cambodia and India, as well as critical examination of extensive literature have shown that only the following four species, instead of 48 nominal species of *Gangesia* and *Silurotaenia* Nybelin, 1942 reported from this region (36 new synonymies proposed), are valid: *Gangesia bengalensis* (Southwell, 1913), type-species of the genus and most common parasite of *Wallago attu* (Siluridae), *G. macrones* Woodland, 1924 typical of *Sperata seenghala* (Bagridae), both species characterized by the possession of two circles of hooks on the rostellum-like organ and several rows of hooklets on the anterior margins of suckers; *G. agraensis* Verma, 1928 from *W. attu* (typical host), which has the scolex with only one circle of hooks and 1–3 incomplete rows of tiny hooklets on the suckers; and *G*. *vachai* (Gupta and Parmar, 1988) n. comb. from several catfishes, which possesses 4–6 circles of hooks and 5–11 rows of hooklets on the anterior half of suckers. Scolex morphology, including surface ultrastructure (microtriches), of all but one species (*G. vachai*) is described for the first time using scanning electron microscopy. A phylogenetic analysis based on the partial sequences encoding the large nuclear ribosomal subunit RNA gene has shown that three Indomalayan species, namely *G. bengalensis*, *G. macrones* and *G*. *vachai*, form a monophyletic group within *Gangesia*, whereas *G. agraensis* tends to form a clade with the Palaearctic species of the genus. A table with differential characters of all species from the Indomalayan Region is also provided together with a key to identification of genera of the subfamily Gangesiinae. The present study demonstrates that species of *Silurotaenia* do not occur in the Indomalayan region.

## Introduction

*Gangesia* was proposed by Woodland [1] to accommodate two species that he described from two catfishes in India, namely *Gangesia wallago* Woodland, 1924 from the silurid *Wallago attu* (Bloch and Schneider, 1801) and *G. macrones* Woodland, 1924 from the bagrid *Sperata seenghala* (Sykes, 1839) (syns. *Mystus seenghala* and *Macrones seenghala*). However, *G. wallago* in fact included two separate species, one conspecific with *Ophryocotyle bengalensis* Southwell, 1913 ( = *Gangesia bengalensis* [Southwell, 1913] Verma, 1928; type-species of the genus) and another one, for which Verma [2] proposed the name *Gangesia agraensis* (synonym *Gangesia wallago* Woodland, 1924 *in part*). Since Woodland [1] used the name *G. wallago* for both taxa, i.e. *G. bengalensis* and *G. agraensis*, Verma’s proposal avoided confusion. His taxonomic action was accepted by subsequent authors, including Freze [3] and Rego [4], but Southwell [5] and Schmidt [6] considered *G. agraensis* to be a synonym of *G. bengalensis*.

Subsequently, a number of species have been described from the same fish hosts, especially from *W*. *attu* and *S*. *seenghala* (see Tables 1–3). However, descriptions of these species were inadequate and were based on apparently decomposed specimens, often with missing hooks due to *post mortem* disintegration of tissues, including detachment of the tegument and surface structures (hooks, hooklets and microtriches) (see, e.g., [7]). In addition, type specimens or vouchers of all but one (*G. sindensis*) taxa are not known to exist and all requests for their loan have remained unanswered, which casts serious doubts upon reliability of these species descriptions.

**Table 1:** Selected morphological characteristics of *Gangesia bengalensis* (Southwell, 1913) and its synonyms.

Characteristics	# *G. bengalensis*	## *G. bengalensis*	*G. lucknowia*	* *G. sindensis*	*G. spinocirrosa*	*G. kashmirensis*	*G. mehamdabadensis*	** *G. indica*	** *G. fotedari*
Scolex width	**325–415**	430	365	344–379 (355–433)	400–410	300	416	250–290	260–370
Size of rostellum-like organ	**125–150×150–190**	–	167	138–172 (138×210)	–	140×520	154×164	110–130×190–210	89–100×100–140
Rostellar hook (No.)	**47–54**	51	50	25 (48–50)	44–50	30	46	24–26	30–48
Hooks in each row (No.)	**24–28+23–26**	–	–	(26–28+20–24)	22–25+22–25	–	–	–	–
Rostellar hook – TL	**33–38**	37–43	39	30 (32–38)	30–40	26	20	10–25 (25–28)	16–24 (30–33)
Rostellar hook – LB	**26–33**	–	–	(23–28)	–	–	–	–	(15–20)
Sucker diameter	**150–190**	100–125	180	132–137 (165–180)	110–120	130	199×198	120–140	70–100 (125–150)
No. of rows of hooklets on suckers	**5–8**	several	9	no hooklets (hooklets present)	may be present	many rows	3–9	–	–
No. of testes	**141–197**	200 or more	130–135	more than 100	–	more than 140	90	100–110	120–134
Relative size of CS (LCS/WS)	**1/4–1/3**	about 1/3	about 1/3	about 1/2 (about 1/3)		about 1/3	–	about 1/3	–
No. of uterine branches on 1 side	**18–25**	10–15	15–17	8–9 (15–20)	14–20	9–14	13–30	18–20	9–15
Host	***Wallago attu***	*W*. *attu*	*Eutropiichthys vacha*	*W. attu*	*W. attu*	*Glyptosternum* sp.	*Mystus tengara*	*W. attu*	*Glyptothorax* sp.

Characteristics	*G*. *paithanensis*	*G*. *aurangabadensis*	*G*. *maharashtrii*	*S*. *tictoi*	*G*. *dharurensis*	*G*. *cirrhinae*	G. *rohitae*	*G. seenghali*	*G*. *ramkaei*	*G. bendsurensis*	*G*. *marathwadensis*	*G*. *jayakwadensis*
Scolex width	816	582	330–350	660	255–530	665	403	252–543	NA	316–704	–	265–1228
Size of rostellum-like organ	336×234	220×270	83–121×156–214	219×280	122–135×214–270	184×282	150×160	160×194–233	NA	97–243×379–398	–	288×333
Rostellar hook (No.)	11	48	40–45	many	35–40	27	60	36–38	35–37	35–47	50–60	44
Hooks in each row (No.)	–	–	–	–	–	–	–	–	NA	–	–	25+29
Rostellar hook – TL	18	35–46	40–46	–	33–40	24–40	10	30–43	NA	10–11	–	25–40
Rostellar hook – LB	–	–	–		–	–	–	–	NA	–	–	–
Sucker diameter	168–263	250–390	170–180	166×255–244	196–210×180–210	155–214×252–277	150×155	146–150	NA	70–218×369–379	–	295–311×265–273
No. of rows of hooklets on suckers	–	–	–	–	no hooklets	–		–	no hooklets	–	–	–
No. of testes	280–300	350–360	170–185	575–590	60–70	100–110	140–150	220–230	170–190	170–190	25–30	300–310
Relative size of CS (LCS/WS)	–	–	about 1/2	–	–	–	–	–	NA	–	–	about 1/4
No. of uterine branches on 1 side	16–18	–	–		–	numerous	–	18–19	no branches	–	–	16–18
Host	*Puntius ticto*	*Sperata seenghala*	*W. attu*	*P*. *ticto*	*W. attu*	*Cirrhinus cirrhosus*	*Labeo rohita*	*S*. *seenghala*	*W. attu*	*W. attu*	*W*. *attu*	*Clarias batrachus*

TL – total length; LB – length of blade; LCS/WS – length of the cirrus-sac/width of the strobila.

# *G*. *bengalensis* – data from the present study.

## *G*. *bengalensis* – data from Verma (1928).

* *G*. *sindensis* – measurements taken from syntypes in parentheses. The number of rows of spines on the suckers and the number of testes are very difficult to count.

** – measurements calculated from Fig. 1 of Gupta and Parmar (1982) and Figs. a, b of Dhar and Majdah (1983) in parentheses.

NA – not available.

Recently, extensive materials of *Gangesia* tapeworms were collected from *W. attu*, *S. seenghala* and *Mystus* spp. from Bangladesh, Cambodia and India. This new, well fixed and properly processed material made it possible to critically assess the taxonomic status of tapeworms from these hosts and to redescribe the species that are considered to be valid. New morphological data, including those on scolex ultrastructure as revealed by scanning electron microscopy (SEM), are provided (see Table 4).

**Table 2:** Selected morphological characteristics of *Gangesia agraensis* Verma, 1928 and its synonyms.

Characteristics	# *G. agraensis*	*G*. *agraensis*	S. *nybelini*	*G. haryanae*	*G*. *sanehensis*	*G. shindei*	*G*. *clariusae*	*G*. *ambikaei*	*G*. *batrachusi*
Scolex width	**305–340**	360	100	418–496	435–566	270–720	262–534	197–827	194–379
Size of rostellum-like organ	**125–135×150–160**	194×258	470×720	110–201×201–217	126–263 ×160–288	200×280	112–12×214–217	30–295×174–394	49–112×112–189
Rostellar hook (No.)	**28–32**	31	–	2–20	22–28	28	17–20	36–37	–
Rostellar hook-length	**24–27**	20–25	–	31	27–46	32	27–29	15–21	13–25
Sucker diameter	**140–175**	125	76×79	134–186×155–186	152–232×123–203	100–140×240	206×180–184	237–311×220–235	150–170×126–141
No. of rows of hooklets on suckers	**2–3**	1–2	–	–	4–5	–	–	–	–
No. of testes	**142–170**	about 100	130–140	60–200	112–184	180–190	85–90	388–400	105–115
Relative size of CS (LCS/WS)	**1/5–2/5**	1/3–1/2	–	–	–	–	–	–	–
VOC/WP	**16–22%**	–	9–12%	–	–	–	–	–	–
No. of uterine branches on 1 side	**14–20**	20–30	–	–	10–24	–	–	–	–
Host	***Wallago attu***	*W. attu*	*Proeutropiichthys taakree*	*W. attu*	*W. attu* and *Cirrhinus cirrhosus*	*Puntius ticto*	*Clarias batrachus*	*W. attu*	*C*. *batrachus*

CS – cirrus-sac; LCS/WS – length of the cirrus-sac/width of the strobila; VOC/WP – width of ventral osmoregulatory canal/width of the proglottis.

# *G*. *agraensis* – data from the present study.

For the first time molecular data of newly collected material of these species were used for confirmation of conspecificity of individual taxa and to assess their phylogenetic relationships.

## Materials and Methods

Tapeworms were collected from wallago *Wallago attu* in Durgapur in Bangladesh in 2011, in Maharashtra, India in 2008, in West Bengal, India in 2008–2011 and in Phnom Penh, Cambodia in 2010; from bagrid catfishes *Mystus* spp. in Assam, India in 2011 and in West Bengal in 2009 and 2011; and from giant river catfish *Sperata seenghala* in Maharashtra in 2010–2012. Fish examined were sold in fish markets and/or from fishermen. This implies they were aimed at human consumption and thus no permission was needed to get these fish that were caught just for commercial purposes. In some cases (big *Wallago attu* catfish), only guts were purchased from fish sellers. Fish that were still alive when examined were killed by dorsal pithing (spinal cord and blood vessels cutting immediately behind the head), which is a method allowed by the law of the Czech Republic, European Directive and local animal care committees.

**Table 3:** Selected morphological characteristics of *Gangesia macrones* Woodland, 1924 and its synonyms.

Characteristics	#G. macrones	*G*. *macrones*	*S. paithanensis*	*S*. *barbusi*	*S*. *macroni*	*S*. *singhali*	*S*. *behairvnathi*	*S. shastri*	*G*. *mastacembali*	*S*. *raoii*
Scolex width	**215–275**	194	273	185	–	240–260	“86”	191–249	284	233–300
Size of rostellum-like organ	**90–115×110–175**	109	167×174	148×194	116×126	160×180	“42×45”	119×169	83×171	87–165×179–247
Rostellar hook (No.)	**38–40+42–45**	33–47	“58”	“many”	“many”	“many”	“30–40”	“80–85”	“18”	“many”
Larger rostellar hook-length	**10–13**	11–15	–	–	–	–	“71”	–	“13–85”	“14–24”
Smaller rostellar hook-length	**5–7**	5–8	–	–	–	–	–	–	–	
Sucker diameter	**80–90**	66–70	91	16–97	–	40–60	“23”	–	40×51	72–87
No. of rows of hooklets on suckers	**4–11**	numerous	–	–	–	–	–	–	–	–
No. of testes	**90–140**	over 100	82–85	135–140	68	“370–390”	“250–260”	72–78	103	125–130
LO/LP	**22–25%**	15–20%	*15–18%	*22%	*18–23%	*17–24%	*21%	*19–22%	–	*25–26%
No. of uterine branches on 1 side	**25–35**	20–30	–	–	–	8–10	–	–	–	–
Host	***Sperata seenghala***	*S*. *seenghala*	*S*. *seenghala*	*Puntius ticto*	*S*. *seenghala*	*S*. *seenghala*	*Mastacembelus armatus*	*S*. *seenghala*	*M*. *armatus*	*S*. *seenghala*

LO/LP – length of the ovary/length of the proglottis.

# *G*. *macrones* – data from the present study.

* – calculated from the figures in original descriptions.

Unreliable data within quotation marks (see Remarks).

Tapeworms, obtained from live or fresh fish, were gently rinsed in 0.9% NaCl solution. A small piece, usually a few posteriormost proglottides, was cut off and placed in molecular-grade ethanol for DNA sequencing (see below). The worms were then placed into a small amount of saline in a beaker or big vial, and hot (boiling) 4% formaldehyde solution ( = formalin) was added, which kept the worms relaxed, not contracted or deformed (see Oros et al., 2010 [8] for more data on this fixation procedure). After 2–3 weeks, formalin was replaced by 70% ethanol for storage before further processing of specimens, i.e. staining, sectioning or preparation for scanning electron microscopical (SEM) observations.

**Table 4:** Selected differential morphological characteristics of four valid species of *Gangesia* from the Indomalayan region, based on the present study.

Characteristics/species	*G. bengalensis*	*G. agraensis*	*G. macrones*	*G. vachai*
Scolex size	295–355×325–415	270–300×305–340	160–220×215–275	210–235×220–245
Rostellar hooks – no. rows	2	1	2	4–6
Rostellar hooks – no. hooks	47–54 (24–28+23–26)	28–32	80–85 (38–40+42–45)	250–450
Rostellar hooks – type	1	1	2	1
Rostellar hooks – total length	36–38	24–27	10–13+5–7	2–5
Rostellum-like organ width (R)	150–190	150–160	110–175	100
Sucker diameter (S)	150–190	140–175	80–90	95–110
R/S ratio	0.96–1.02 (x = 0.99)	0.85–1.06 (x = 0.97)	1.27–1.95 (x = 1.53)	0.91–1.05 (x = 0.98)
No. rows of hooklets on suckers	5–8	2–3	9–12	4–11
Types of filitriches on suckers	mostly acicular and few capilliform	papilliform	capilliform	unknown
Layers of testes	2–3 incomplete	2–3 incomplete	single	single
LO/LP	1/3 i.e. 33–41% (x = 36%)	1/3 i.e. 31–37% (x = 34%)	1/4 i.e. 22–25% (x = 24%)	1/3 i.e. 30–37% (x = 33%)
VOC/WP	4–11%	16–22%	3–4%	5–6%
Position of vitelline follicles	mostly medullary, few paramuscular	all medullary	mostly medullary, few paramuscular	mostly medullary, few paramuscular
Typical host	*Wallago attu*	*Wallago attu*	*Sperata seenghala*	catfishes

x = mean; LO/LP – length of ovary/length of proglottis;VOC/WP – width of ventral osmoregulatory canal/width of proglottis.

For light microscopy, specimens were stained with Mayer’s hydrochlorid carmine, destained in 70% acid ethanol (i.e. ethanol with several drops of HCl), dehydrated through a graded ethanol series, cleared in clove oil (eugenol), and mounted in Canada balsam as permanent preparations [9]. Pieces of strobila and scoleces were embedded in paraffin wax, sectioned at 12–15 µm (cross sections of strobila and longitudinal sections of scoleces), stained with Weigert’s haematoxylin and counterstained with 1% acidic eosin B solution [10]. Illustrations were made using a drawing attachment of an Olympus BX51 microscope with the use of Nomarski interference contrast. Measurements were taken using Olympus Image-Pro programme. Eggs from the uterus of unstained, unmounted worms were measured and illustrated in the water. For SEM observations, specimens were dehydrated through a graded ethanol series, transferred to hexamethyldisilazane (HMDS – see [11]), dried in air, sputtered with gold (approximately 10 nm thick) and examined with a Jeol JSEM 7401F microscope.

Newly collected specimens have been deposited in the following collections (for accession numbers – see redescriptions of individual taxa): The Natural History Museum, London, UK (acronym BMNH); Helminthological Collection, Institute of Parasitology, České Bude˘jovice, Czech Republic (IPCAS); Natural History Museum, Geneva, Switzerland (MHNG-PLAT); and U. S. National Parasite Collection, Beltsville, USA (USNPC). Specimens intended to be deposited at the Zoological Survey of India, Kolkata (Calcutta), India (ZSI) could not be accessioned because of the absence of a curator of cestode collection (pers. comm. of collection staff to T.S. and P.K.K.).

Type and voucher specimens of the following species of *Gangesia* were examined: *G. macrones* Woodland, 1924 (syntypes – BMNH 1927.8.10.3 and 1964.12.15.246–255), *G. wallago* Woodland, 1924 (syntypes – BMNH 1927.8.10.1–2 and 1964.12.15.256–280), and *G. sindensis* Rehana and Bilqees, 1971 from *W. attu*, Gharo, Pakistan, 12.2.1972 (not designated explicitly as types but in fact representing syntypes – BMNH 1982.5.13.27).

All written requests for loan of the type and voucher specimens of other species of *Gangesia* described from the Indian subcontinent between 1974 and 2011 ([7], [12], [13], [14], [15], [16], [17], [18], [19], [20], [21], [22], [23], [24], [25], [26], [27], [28], [29], [30], [31], [32], [33], [34], [35], [36], [37], [38], [39], [40], [41]) have never been answered and it is thus uncertain whether type specimens or vouchers of any of these species were actually deposited.

In morphological descriptions, measurements are usually those taken from mature segments (only if the number of mature proglottides was too low, measurements were taken from the first pregravid proglottides) and are in micrometres (µm) unless otherwise stated; classification of microtriches follows Chervy [42]. The scientific and common names of fish hosts follow the FishBase online database [43]. Abbreviations of the terms used in redescriptions are as follows: n = number of measurements; L/W = length/width ratio; O/P = ratio of the width of the ovary and width of the proglottis; C/P = ratio of the length of the cirrus-sac and width of the proglottis; R/S = ratio of the diameter of a rostellum-like organ and diameter of the suckers.

Twenty-eight new sequences of the large subunit nuclear ribosomal RNA gene (lsrDNA or 28S rDNA) of 9 nominal taxa were characterized with focus on the Indomalayan *Gangesia* species but also included other genera of the Gangesiinae (see Table 5). Genomic DNA was extracted using a standard phenol-chloroform protocol [44] from 96% ethanol preserved samples. The D1–D3 lsrDNA region was amplified by PCR using the primers and conditions described in Brabec et al. [45]. All products were verified on a 1% agarose gel and purified using exonuclease I and shrimp alkaline phosphatase enzymes [46]. BigDye® Terminator v3.1 cycle sequencing kit and PRISM 3130xl automatic sequencer (Applied Biosystems) were used for bidirectional sequencing of the PCR products using the set of PCR and internal sequencing primers (see [45]). Newly characterized sequences were deposited in the GenBank under the Accession Nos. JX477427–JX477454. Sequences were assembled and inspected for errors in Geneious Pro 5.3.6 [47], aligned using the E-INS-i algorithm of the program MAFFT [48] and the ambiguously aligned positions were manually excluded from resulting alignments in MacClade 4.08 [49]. The phylogenetic relationships were evaluated under the maximum likelihood (ML) criteria in the program RAxML 7.2.8 [50], [51], employing the GTR+Γ substitution model. All model parameters and bootstrap nodal support values (1000 repetitions) were estimated using RAxML. An alternative hypothesis than the one represented by the topology of the best ML tree was tested using the approximately unbiased (AU) test implemented in the program Consel 0.20 ([52], [53]). ML branch lengths of the constrained topology as also the per-site log-likelihood values of both unconstrained and constrained trees were computed in RAxML using the ML settings described above. Subsequently, *p* values of different likelihood-based tests were calculated with Consel.

**Table 5:** List of specimens sequenced.

Specimen	Host	Field No.	Locality	Accession no.	Source
*Acanthobothrium parviuncinatum*	*Urobatis maculatus*	–	Baja California Sur, Mexico	EF095264	[76]
*Electrotaenia malopteruri*	*Malapterurus electricus*	INVE33995	El Minia, Egypt	JX477434	present study
*Gangesia agraensis*	*Wallago attu*	AA28b	Balurghat, West Bengal, India	JX477430	present study
*Gangesia agraensis*	*Wallago attu*	AA82a	Balurghat, West Bengal, India	JX477431	present study
*Gangesia agraensis*	*Wallago attu*	IND166	Balurghat, West Bengal, India	JX477435	present study
*Gangesia agraensis*	*Wallago attu*	IND167	Balurghat, West Bengal, India	JX477436	present study
*Gangesia agraensis*	*Wallago attu*	IND795	Guwahati, Assam, India	JX477439	present study
*Gangesia agraensis*	*Wallago attu*	IND814	Guwahati, Assam, India	JX477440	present study
*Gangesia agraensis*	*Wallago attu*	MH62	Siddeshwar reservoir, Maharashtra, India	JX477443	present study
*Gangesia agraensis*	*Wallago attu*	VNT373	Phnom Penh, Cambodia	JX477454	present study
*Gangesia bengalensis*	*Wallago attu*	AA125a	Rishra, West Bengal, India	JX477427	present study
*Gangesia bengalensis*	*Wallago attu*	AA133	Rishra, West Bengal, India	JX477428	present study
*Gangesia bengalensis*	*Wallago attu*	AA133a	Rishra, West Bengal, India	JX477429	present study
*Gangesia bengalensis*	*Wallago attu*	IND339	Berhampur, West Bengal, India	JX477438	present study
*Gangesia macrones*	*Sperata seenghala*	BAN61	Mymensingh, Bangladesh	JX477433	present study
*Gangesia macrones*	*Sperata seenghala*	MS19	Godavari River, Maharashtra, India	JX477444	present study
*Gangesia macrones*	*Sperata seenghala*	MS20	Godavari River, Maharashtra, India	JX477445	present study
*Gangesia macrones*	*Sperata seenghala*	MS22a	Godavari River, Maharashtra, India	JX477446	present study
*Gangesia parasiluri*	*Silurus asotus*	–	Lake Suwa, Japan	AF286935	[77]
*Gangesia vachai*	*Wallago attu*	BAN186	Durgapur, Bangladesh	JX477432	present study
*Gangesia vachai*	*Mystus* cf. *tengara*	IND303	Siliguri, West Bengal, India	JX477437	present study
*Gangesia oligonchis*	*Tachysurus fulvidraco*	RUS29a	Ilistaya River, Far East, Russia	JX477448	present study
*Gangesia oligonchis*	*Tachysurus fulvidraco*	RUS29b	Ilistaya River, Far East, Russia	JX477449	present study
*Gangesia oligonchis*	*Tachysurus fulvidraco*	RUS32	Ilistaya River, Far East, Russia	JX477450	present study
*Gangesia oligonchis*	*Tachysurus fulvidraco*	RUS32b	Ilistaya River, Far East, Russia	JX477451	present study
*Gangesia oligonchis*	*Tachysurus fulvidraco*	RUS85b	Ilistaya River, Far East, Russia	JX477452	present study
*Postgangesia inarmata*	*Silurus glanis*	INVE 34212	Tigris River, Mosul, Iraq	AM931032	[78]
*Postgangesia inarmata*	*Silurus glanis*	IRQ33	Lesser Zab, Iraq	JX477441	present study
*Postgangesia inarmata*	*Silurus glanis*	IRQ34	Lesser Zab, Iraq	JX477442	present study
*Ritacestus ritaii*	*Rita rita*	IND067	Malda, West Bengal, India	JX477447	present study
*Silurotaenia siluri*	*Silurus glanis*	–	Orlík reservoir, Czech Republic	AJ388592	[79]
*Vermaia pseudotropii*	*Clupisoma garua*	IND62b	Mukutmanipur, West Bengal, India	JX477453	present study

## Results

### Species Composition and Differential Criteria

A critical review of literature (papers from years 1948–2012) has shown that: (1) most specimens used for descriptions of new taxa were decomposed; (2) descriptions were incomplete; e.g., cross sections were always missing, data on the number of uterine branches were often omitted, etc.; (3) many data were undoubtedly erroneous and individual structures were misinterpreted, e.g., vitelline follicles were counted as testes and muscle cells were considered to be hooks, which were in fact lost due to detachment of the tegument; (4) data in the text did not correspond to those inferred from illustrations, which were always very schematic and incomplete; (5) species were differentiated on the basis of questionable taxonomic characters, such as negligible differences in the number of testes (often erroneous) and unspecified shape of the cirrus-sac; (6) types are not known to exist and never were available upon request for all but three species of *Gangesia* and *Silurotaenia* described from the Indian subcontinent; and (7) identification of some fish hosts is questionable.

To clarify this confused situation, the following approach was applied (see also [54]): (1) new, well fixed material was compared with the oldest available descriptions to avoid other inflation of new names in the literature; and (2) the following key taxonomic (differential) characteristics were selected to assess the validity of inadequately described species (see Table 4 and Remarks on individual species): (i) number of rows of rostellar hooks and their size; (ii) number of rows of hooklets on the suckers; (iii) ratio of the width of the rostellum-like organ and diameter of the suckers; (iv) relative length of the ovary, i.e. ratio of its length to the length of proglottides; (v) width of the scolex; (vi) diameter of the suckers; and (vii) relative width of ventral osmoregulatory canals, i.e. ratio of their width to the width of the proglottis.

Examination of newly collected material has shown that only four species, instead of 48 nominal taxa, of *Gangesia* and *Silurotaenia*, namely *Gangesia bengalensis* (Southwell, 1913) (type-species of the genus), *G. agraensis* Verma, 1928, both parasitizing most commonly *Wallago attu*; *G. macrones* Woodland, 1924, a typical parasite of *Sperata seenghala*; and *G*. *vachai* (Gupta and Parmar, 1988) n. comb., from catfishes of several families, are valid. Their redescriptions based on type materials, if available, and newly collected specimens are provided below. The taxonomic status of some species, the descriptions of which were not available, could not be clarified (see “Taxa of unclear status”).

### Survey of Species

#### *Gangesia bengalensis* (Southwell, 1913) Verma, 1928

Synonyms (in chronological orders): *Ophryocotyle bengalensis* Southwell, 1913; *Gangesia wallago* Woodland, 1924 (*in part*); *Gangesia lucknowia* Singh, 1948; new synonyms and invalid names: *G*. *sindensis* Rehana and Bilqees, 1971; *G*. *spinocirrosa* Rehana and Bilqees, 1973; *G*. *kashmirensis* Dhar and Fotedar, 1979; *G*. *etawaensis* Malhotra, Dixit and Capoor, 1980 (*nomen nudum*); *G*. *mehamdabadensis* Malhotra, Dixit and Capoor, 1980; *G*. *indica* Gupta and Parmar, 1982; *G*. *fotedari* Dhar and Majdah, 1983; *G*. *paithanensis* Jadhav, Shinde and Kadam, 1983; *G*. *aurangabadensis* Shinde and Wankhede, 1990; *G*. *maharashtrii* Hiware and Jadhav, 1995; *G*. *attui* Chavan, 1997 (*nomen nudum*); *G. dharurensis* Jadhav and Tat, 1997; *G*. *parbhaniensis* Chavan, 1997 (*nomen nudum*); *G*. *cirrhinae* Patel, Shinde and Khan, 1999; *G*. *rohitae* Shinde, Mahajan and Begum, 1999; *G*. *seenghali* Hiware, 1999; *G*. *rohitae* Pawar, Lakhe, Shinde and Patil, 2004 (homonym of *G*. *rohitae* Shinde, Mahajan and Begum, 1999); *G*. *ramkaei* Pawar and Hiware, 2008; *G*. *bendsurensis* Reddy, Wankhede, Dhole and Anand, 2011; *G*. *marathwadensis* Bhure, Nanware and Dhondge, 2011; *G*. *jayakwadensis* Bhavara and Shukla, 2012; *Silurotaenia tictoi* Shinde, Kadam and Jadhav, 1984.

Redescription (based on 24 specimens from *W*. *attu* from India, including four scoleces observed using SEM): Strobila with acraspedote proglottides, more than 47 mm long (n = 3; 30–50 mm according to Verma, 1928 [2]) and up to 880 wide (800 according to Verma, 1928 [2]). Proliferative zone 3.3–4.3 mm long and 225–320 wide. Strobila consists of more than 164 proglottides (n = 4): 115–150 immature (up to appearance of spermatozoa in vas deferens), only 3–4 mature (up to appearance of eggs in uterus), 5–10 pregravid and gravid (actual number is higher because posteriormost proglottides were cut for molecular analyses). Proglottides variable in shape, usually slightly wider than long; mature proglottides 560–765 long and 720–870 wide (n = 8).

**Figure 1:**
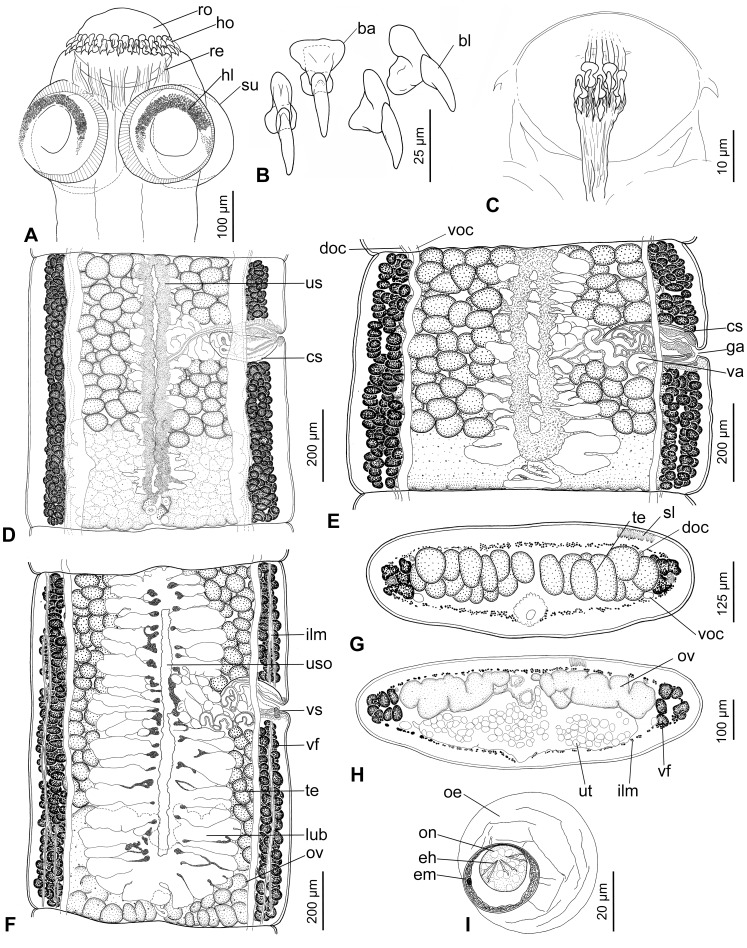
*Gangesia bengalensis* (Southwell, 1913) from *Wallago attu*, India. A. Scolex, dorsoventral view (MHNG-PLAT 82308, field no. AA 133B). **B.** Rostellar hooks (IPCAS C-616, field no. AA 133). **C.** Detail of retractor muscles. **D**, **E.** Mature proglottides, ventral view (IPCAS C-616, field no. AA 133 and MHNG-PLAT 60721, field no. 147/08). **F.** Gravid proglottis, ventral view (IPCAS C-616, field no. AA 133). **G**, **H.** Cross sections at level of testicular field and ovary, respectively (IPCAS C-616, field no. AA 133); note that subtegumental layer is not fully illustrated. **I.** Egg drawn in distilled water. Abbreviations: **ba** – base of hook, **bl** – blade of hook, **cs** – cirrus-sac, **doc** – dorsal osmoregulatory canal, **eh** – embryonic hook, **em** – embryophore, **ga** – genital atrium, **hl** – hooklets, **ho** – hooks, **ilm** – internal longitudinal muscles, **lub** – lateral uterine branch, **oe** – outer envelope, **on** – oncosphere, **ov** – ovary, **re** – retractor muscles, **ro** – rostellum-like organ, **sl** – subtegumental layer, **su** – sucker, **te** – testes, **us** – uterine stem, **uso** – uterine slit-like opening, **ut** – uterus, **va** – vagina, **vf** – vitelline follicles, **voc** – ventral osmoregulatory canal, **vs** – vaginal sphincter.

**Figure 2:**
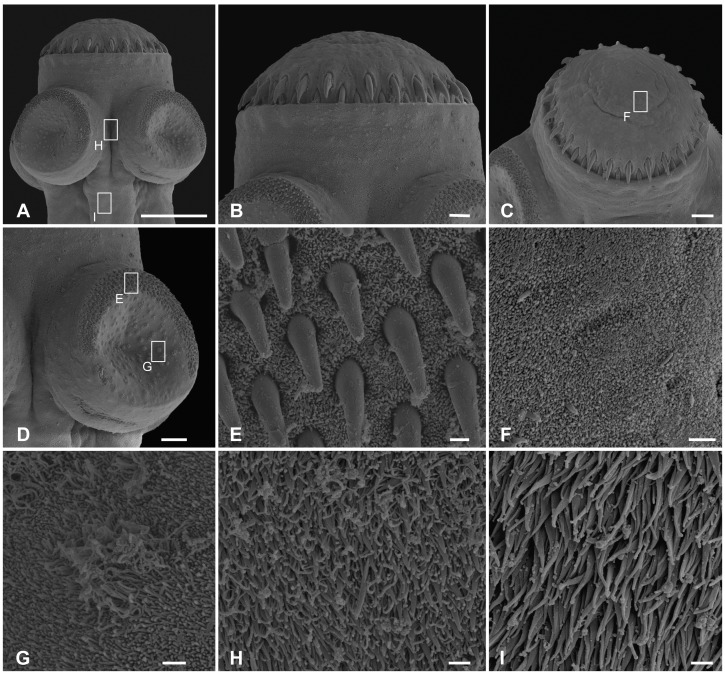
Scanning electron photomicrographs of *Gangesia bengalensis* (Southwell 1913). **A.** Scolex, dorsoventral view. **B, C.** Detail of rostellum-like organ; note two rows of rostellar hooks. **D.** Sucker with small hooklets on the outer rim. **E.** Detail of the outer rim of the sucker with hooklets. **F.** Detail of short, dense acicular filitriches on the rostellum-like organ. **G.** Detail of acicular filitriches and few capilliform filitriches on the sucker. **H.** Detail of capilliform filitriches and small gladiate spinitriches in between the suckers. **I.** Detail of gladiate spinitriches on the neck region. Scale bars: **A** –100 µm; **B** –10 µm; **C**, **D** –20 µm; **E**–**I** –1 µm.

Scolex wider than neck, 295–355 long by 325–415 wide (n = 9). Rostellum-like organ 125–150 long and 150–190 wide, armed with two, alternating rows of hooks; total number of hooks 47–54 (n = 9; upper row composed of 24–28 hooks, lower row of 23–26 hooks). Hooks in both rows similar in total length, 36–38 long (n = 40; blade 26–33), but hooks of upper row with massive basal plate, much wider in anterior part (maximum width 15–17), with deep constriction in middle, whereas hooks of lower row with narrow basal plate, widened posteriorly (maximum width 10–13) (Fig. 1B). Suckers four, uniloculate, 150–190 in diameter (n = 30); R/S = 0.98–1.02. Outer rim of anterior 1/2–1/3 of suckers covered by minute (3–4 long) hooklets, nearly 400 in total, arranged in 5–8 (mostly 7; n = 25) rows (Figs. 1A; 2A,D).

Powerful retractor muscle bundles surround rostellum-like organ, joined each other at level of suckers, forming wide band of muscles (retractors) at neck region (Figs. 1A; 9B).

Rostellum and rim of suckers uniformly and densely covered with short acicular filitriches (Fig. 2E,F); tufts of capilliform filitriches present within sucker cavity (Fig. 2G). Both capilliform filitriches and gladiate spinitriches present between suckers (Fig. 2H). Neck region covered with gladiate spinitriches (Fig. 2I).

Inner longitudinal musculature well developed, anastomosed, forming isolated bundles of muscle fibres. Subtegumental muscles well developed. Ventral osmoregulatory canals thin-walled, their width representing 4–11% of width of mature proglottides, overlapping lateralmost testes, may form transverse commissures and/or anastomoses in some proglottides. Dorsal osmoregulatory canal narrow, thick-walled, may reach vitelline follicles laterally (Fig. 1D,E).

Testes medullary, in one field, oval, 65–80 long and 50–65 wide (n = 30), numbering 141–197 (n = 6), mostly forming two or three incomplete layers (Fig. 1D–G), occupying 2/3–3/4 of proglottis length. Cirrus-sac elongate, thick-walled, 190–245 long by 75–105 wide (n = 15; L/W ratio = 2.1–3.0); C/P = 25–35%, i.e. 1/4–1/3 of proglottis width. Genital pore irregularly alternating, pre-equatorial, situated at 28–37% (n = 15) of proglottis length from anterior margin.

Ovary medullary, bilobed, 490–665 wide (n = 6), each lobe almost rectangular with numerous lobules extending both dorsally and ventrally; ovary 180–315 long, occupying more than 1/3 (33–40%) of length of mature proglottides (Fig. 1D,E); O/P = 69–76% (n = 6). Mehlis’ gland 60–75 in diameter (n = 6), representing 8–10% of proglottis width. Vagina thick-walled, vaginal canal may be coiled in distal part (Fig. 1E,F), posterior (28%) or anterior (72%; n = 64) to cirrus-sac, with higher concentration of chromophilic cells in its distal (terminal) part, and ring-like vaginal sphincter near genital atrium (Fig. 1E,F).

Vitelline follicles medullary with some follicles paramuscular (penetrating between muscle fibres of inner longitudinal musculature), in two longitudinal bands on both sides of proglottis, occupying almost its total length (95–99% on poral side, 94–99% on aporal side in mature proglottides; n = 10); bands interrupted at level of terminal genitalia on ventral side (Fig. 1D–F), with few follicles on dorsal side.

Uterus medullary, with development of type 1 according to de Chambrier et al. [55], defined as follows: in immature proglottides, uterine stem present as longitudinal concentration of chromophilic cells along median line. Lumen of uterus appears in last premature proglottides, gradually extending to form thick-walled tubular structure. Eggs appear simultaneously with formation of lateral, thin-walled diverticula. In pregravid proglottides, lateral diverticula remain thin-walled, 18–25 in number on each side (n = 20), occupy up to 62% of proglottis width (Fig. 1F), may partially overlap ovary (Fig. 1F). Uterus with slit-like openings (Fig. 1F).

Eggs with hyaline, spherical outer envelope, 50–55 in diameter (measured in eggs liberated from uterus in distilled water; n = 30); embryophore thick (3–4 wide), spherical, 25–30 in diameter, consisting of two layers; outer layer thinner than nuclei-containing envelope; oncosphere spherical, 15–20 in diameter, with three pairs of embryonic hooks, 7–8 long (Fig. 1I).

*Type-host*: *Channa striata* (Bloch, 1793) (Perciformes: Channidae) or *Labeo rohita* (Hamilton, 1822) (Cypriniformes: Cyprinidae) (not explicitly mentioned in the original description).

*Other hosts*: *Wallago attu* (Bloch and Schneider, 1801) (Siluriformes: Siluridae; most common host); *Eutropiichthys vacha* (Hamilton, 1822) (Siluriformes: Schilbeidae); *Cirrhinus cirrhosus* (Bloch, 1795); *Barbus ticto* (Hamilton, 1822) (both Cypriniformes: Cyprinidae); *Mystus tengara* (Hamilton, 1822); *Sperata seenghala* (Sykes, 1839) (both Siluriformes: Bagridae); *Glyptothorax* sp. (Siluriformes: Sisoridae).

*Type-locality*: Berhampur Court, West Bengal, India.

*Material studied*: 15 specimens from *W. attu* from the Hooghly River in Rishra (field No. AA 125– collected on 14. iii. 2008; AA 133–27. iii. 2008) and the Bhagirathi River in Berhampur (IND 339–12. iii. 2009), all West Bengal, India, collected by A.A., T.S. and P.K.K. (BMNH 2012.8.23.4–5; IPCAS C-616; MHNG-PLAT 82308, 82309; USNPC 105944; ZSI – not accessioned); three specimens from *W. attu* from the Brahmaputra in Guwahati, Assam, India (IND 795–17. iii. 2011; IND 814–18. iii. 2011), collected by A.A., T.S. and M.O. (IPCAS C-616); three specimens from *W. attu* from fish market in Gangapur, Maharashtra, India (147/08– on 21. iii. 2008), collected by M.O. (MHNG-PLAT 60721); and six specimens from *W. attu* from Masoli dam lake and Yeldari dam lake on the Purna River, Maharashtra, India (MH 28, 39, 60, 62, 63–8. ix. 2007, 8. i. 2008, and 20. viii. and 20. x. 2010), collected by S.P.C. (IPCAS C-616; MHNG-PLAT 75463, 82307; ZSI – not accessioned).

*Site of infection*: intestine.

*Prevalence*: 100% (n = 5, i.e. five fish examined) in Rishra, West Bengal; 100% (n = 1) in Berhampur, West Bengal; 20% (n = 5) in Gangapur, Maharashtra.

*Intensity of infection*: 1–7 tapeworms (mean 2.6) in Rishra, West Bengal; one tapeworm in Berhampur, West Bengal.

*Distribution*: India (Assam, Gujarat, Haryana, Kashmir, Maharashtra, Uttar Pradesh, West Bengal), Pakistan and Sri Lanka.

*Remarks*: Southwell [56] described this species as *Ophryocotyle bengalensis*, but Woodland [1] did not accept its validity because of its insufficient description (see footnote on p. 447 in his paper). Instead, he proposed a new species, *Gangesia wallago*, as the type species of his new genus *Gangesia*, even though he admitted conspecificity of *G. wallago* and *O. bengalensis* (“Southwell (1913*a*, *b*), in describing what were almost certainly examples of the *G. wallago* ^1^ I have described above, …” – see p. 447 in [1]).

**Figure 3:**
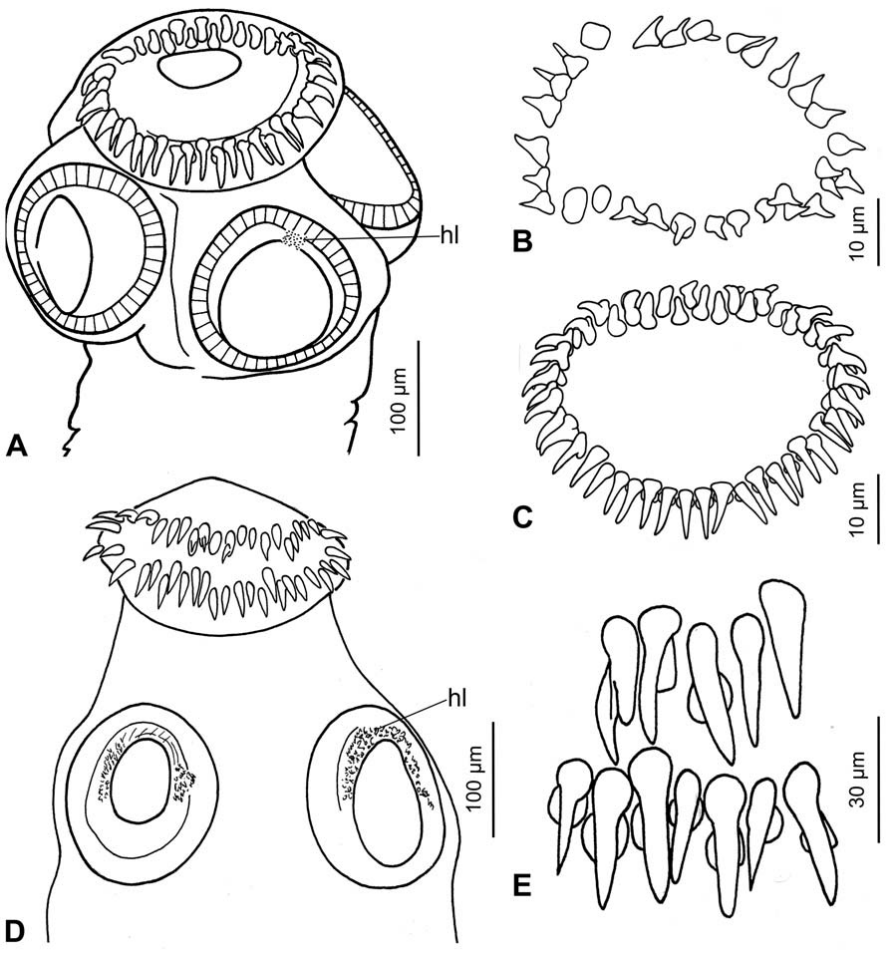
Type specimens of *Gangesia wallago* Woodland, 1924 and *G*. *sindensis* Rehana and Bilqees, 1971. A. Scolex of *G*. *wallago*, sub-apical view; note presence of two irregular rows of rostellar hooks ( = *G. bengalensis*) and hooklets (hl) on the sucker. **B**, **C.** Details of arrangement of hooks on a rostellum-like organ of *G*. *wallago* (B – *G. agraensis*; C – *G. bengalensis*); note two types of arrangement. **D.** Scolex of *G*. *sindensis*, dorsoventral view; note the presence of hooklets on the suckers. **E.** Details of arrangement of hooks on a rostellum-like organ of *G*. *sindensis* ( = *G. bengalensis*); note the presence of a double rows of hooks.

Woodland [1] in fact studied two separate species, one corresponding to *O. bengalensis*, whereas the other representing undescribed species, for which Verma [2] proposed the name *Gangesia agraensis*. Examination of type specimens of *G. wallago* by the present authors confirmed that Woodland [1] actually described two different species under the name *G. wallago* (see Fig. 3A–C). Southwell [5] listed *Gangesia bengalensis* as the type species of the genus, but synonymized *G*. *agraensis* with *G*. *bengalensis*, which was then accepted by some authors (e.g. Yamaguti [57], Schmidt [6]).

Newly collected material from India (Assam, Maharashtra and West Bengal) enabled us to redescribe *G. bengalensis*, which is typified by the following morphological characteristics: (i) the rostellum-like organ is armed with two rows of hooks of a similar length (36–38 µm long), 47–54 in number (upper row with 24–28 and lower row with 23–26 hooks), with broader hooks in the anterior (upper) circle; (ii) the outer rim of the anterior half of the suckers is covered with minute (length 3–4 µm) hooklets arranged in 5–8 rows (Figs. 1A; 2A,D); (iii) base of the hooks of the upper row consists of a massive base plate, much wider in the anterior part, with deep constriction in the middle, whereas the hooks of the lower row possess a narrow base plate widened posteriorly; (iv) vitelline follicles are medullary, with a few follicles paramuscular, i.e. penetrating between the inner longitudinal musculature (Fig. 1G,H); (v) cavity of suckers covered with acicular filitriches, with few tufts of capilliform filitriches; (vi) ventral osmoregulatory canals occupy 4–11% of the width of mature proglottides; (vii) the uterus possesses slit-like openings in pregravid proglottides.

The type host of *G*. *bengalensis* was not designated by Southwell [56], who listed snakehead murrel *Channa striata* (Channidae) and rahu *Labeo rohita* (Cyprinidae) as the definitive hosts of *O*. *bengalensis*. However, the former fish host was heavily infected, whereas tapeworms in *L. rohita* were few in numbers [56]. Southwell [58] then reported *Wallago attu* as an additional fish host. The absence of reliable records of *G. bengalensis* from *C*. *striata* and *L*. *rohita* indicates that these fishes were most probably only atypical or accidental hosts. The present authors dissected 32 specimens of *C*. *striata* from Bangladesh, India and Indonesia, and 12 specimens of *L*. *rohita* from Bangladesh and India but have never found any proteocephalidean cestode.

Except for *G*. *wallago* and *G*. *sindensis*, type material of any species of *Gangesia* described from *W. attu* and other fish hosts from India and Pakistan has not been available and it is not known to exist. Consequently, the validity of all species had to be assessed solely by comparison of the data from the original descriptions, which, however, suffered from many flaws and deficiencies (see below). As a result, most synonymies proposed in this paper rely on the characteristics considered to be diagnostic (species-specific) as listed above. In contrast, differential criteria used to distinguish allegedly new species of *Gangesia* and/or *Silurotaenia* were found to be doubtful and unreliable, which justifies proposed invalidation of all the species discussed below. The present authors are aware that such arbitrarily taken decision does not exclude the possibility that some of the taxa synonymized with *G. bengalensis* are valid, but their validity must be justified by reliable and convincing arguments based on adequate morphological descriptions of well-fixed specimens that are deposited in institutional collections and available upon request.

Singh [59] described *Gangesia lucknowia* from the schilbeid catfish *Eutropiichthys vacha* from Lucknow, Uttar Pradesh, India. The species was characterized by the possession of two rows of rostellar hooks of the same size and shape, which is also a typical feature of *G. bengalensis*. In addition, most differential characteristics overlap or are identical in both species (see Table 1 and the redescription of *G. bengalensis* above). Consequently, we accept Malhotra et al.’s [16] conclusion that *G. lucknowia* is a synonym of *G. bengalensis*.

Rehana and Bilqees [60] proposed a new species, *G*. *sindensis*, on the basis of two partially digested, strongly flattened and thus deformed specimens found in *W. attu* from Pakistan (see Fig. 1A–E in Rehana and Bilqees [60]). The main distinguishing features of the species were the alleged absence of hooklets on the suckers, number and size of the rostellar hooks (single row) and the number of uterine diverticula. However, examination of the existing syntypes (though designated as vouchers) of *G. sindensis* (BMNH 1982.5.13.27) has shown that suckers are in fact covered with hooklets (see Fig. 3D) and that there are two rows of rostellar hooks (see Fig. 3D,E and Table 1). Hence, *G*. *sindensis* is synonymized with *G*. *bengalensis*.

The same authors [61] described another new species, *G*. *spinocirrosa*, from the same host (*W*. *attu*) and locality from Pakistan, but this original description was not available to the present authors. However, R. Rehana provided a detailed description of *G*. *spinocirrosa*, including illustrations, in her PhD thesis [62]. She distinguished this species from congeners by the possession of a spiny cirrus. However, it is obvious from Figs. 106 and 107 in Rehana [62] that the worms were decomposed and muscles fibres of the cirrus were misinterpreted as spines. Otherwise, all other characters including the arrangement and number of hooks correspond to those of *G*. *bengalensis* (see Table 1). Therefore, *G*. *spinocirrosa* becomes a junior synonym of *G*. *bengalensis*, too.

Dhar and Fotedar [7] described *G*. *kashmirensis* from a catfish identified as *Glyptosternum* sp. from Kashmir, India and distinguished the new species from *G*. *bengalensis* (based on a very brief species diagnosis provided by Southwell [56]) by several characters, such as shape of the scolex, number of rows of rostellar hooks, number of testes, etc. (see Table I of Dhar and Fotedar [7]). In fact, *G*. *kashmirensis* does not differ from *G*. *bengalensis* as redescribed herein, which is obvious from comparison of their morphology and measurements (see Table 1), and it is also synonymized with this species.

*G*. *mehamdabadensis* was described by Malhotra et al. [15] from *Mystus tengara* (misspelled as *M*. *tengra* in the original paper) from Gujrat, India. Morphological description seems to be comprehensive, but cross sections were not provided (even though they were mentioned in the Material & Methods section). The taxon is in fact identical with *G. bengalensis* in all but one characteristic, the only alleged difference being the arrangement of the hooks on the rostellum-like organ in one, instead of two rows. However, the specimens decribed by Malhotra et al. [15] were strongly flattened, as obvious from their Fig. 1. It has been observed by the present authors that double rows of hooks may appear as a single one in strongly flattened worms. Malhotra et al. [15] reported the holotype of *G. mehamdabadensis* to have been deposited in the Commonwealth Agricultural Bureaux Institute of Parasitology in St. Albans, UK, but no specimens are now in The Natural History Museum in London, where this collection is currently placed (E. Harris, Curator – pers. comm.). It is thus not possible to reliably count the number of rows of hooks in the species, which is invalidated as a junior synonym of *G. bengalensis*. In the same paper [15], a new species, *Gangesia etawaensis*, was mentioned, but it was apparently an error and *G. mehamdabadensis* ( = *G. bengalensis*) was incorrectly named. Since no description of the former species appeared in that article, *G. etawaensis* becomes a *nomen nudum*.

Gupta and Parmar [17] described *G*. *indica* from *Wallagonia attu* ( = *Wallago attu*) from Lucknow, India. Their new species is mainly characterised by the presence of 24–26 rostellar hooks arranged in two rows and suckers without hooklets. However, this does not correspond to figures: 12+13 hooks, i.e. 25 in total, are illustrated, but just on one side, which means that the total number of hooks is about 50. The hooks are of the same type, not of two types as written in the text. The hooks illustrated in Fig. 1 of Gupta and Parmar [17] are larger (length 25–28 µm) than mentioned in the text (length 10–25 µm only). Thus the species is in fact indistinguishable from *G*. *bengalensis* with which is synonymized.

*Gangesia fotedari* was described by Dhar and Majdah [18] from a catfish tentatively identified as *Glyptothorax* sp. from Kashmir, India. This cestode was differentiated from *G*. *kashmirensis* Dhar and Fotedar, 1979 ( = *G*. *bengalensis*; see above) by very few characters, namely the number of hooks and their size, and the size of the scolex (see Table 1). However, these allegedly differential characters should be considered with caution, because the characteristics reported in the morphological description by Dhar and Majdah [18] do not correspond to those illustrated in figures (see Table 1). Based on general resemblance of most morphological characteristics, *G*. *fotedari* is synonymized with *G*. *bengalensis* until adequate redescription of the former species, based on good quality material, confirms validity of this poorly described taxon.

Jadhav et al. [19] described *G*. *paithanensis* from *Barbus ticto* ( = *Puntius ticto*) from the Godavari River at Paithan, India. The description was very brief and incomplete (e.g., no data on eggs were provided) but it is evident that the specimens were digested and hooks detached due to decomposition (see Figs. A and B of Jadhav et al. [19]). Low number of the hooks (only 11!) reported in the paper is apparently incorrect. The authors observed two types of hooks, which is a characteristic typical of *G*. *bengalensis*, and their length was similar (16–18 µm). Other morphological features of *G*. *paithanensis* also correspond to those of *G*. *bengalensis* (see Table 1) and thus *G*. *paithanensis* is synonymized with the former species.

The same authors [21] described *Silurotaenia tictoi* from the same fish host (*Puntius ticto*) and locality (Paithan), but this description was based on completely digested material with all hooks lost (see Plate 4A in Shinde et al. [21]). It is evident from Plates 4B and 4C that the authors misinterpreted the muscles in the rim of the rostellum-like organ as hooks and vitelline follicles as testes. Morphological description was incomplete and illustrations erroneous (data on the uterus were missing and the uterus was not illustrated in Plate 4C, etc.). Type specimens of this species never existed (B. Jadhav – pers. comm.), but a large size of the scolex and relatively narrow ventral osmoregulatory canals indicate that *G. paithanensis* is conspecific with *G*. *bengalensis*, with which it is synonymized.

*G*. *aurangabadensis* was described by Shinde and Wankhede [25] from macerated specimens (see Fig. 1 of Shinde and Wankhede [25]) allegedly found in *Macrones seenghala* ( = *Sperata seenghala*) from Paithan, Maharashtra, India. Morphological description was incomplete (e.g., no data on uterine branches and vitelline follicles were provided – see Fig. 1C), but it is evident that *G*. *aurangabadensis* is conspecific with *G*. *bengalensis* (see Table 1).

Hiware [29] described *G*. *seenghali* from *Mystus seenghala* ( = *Sperata seenghala*) from Satara, Maharashtra, India. Description of this species is very poor (e.g., vitelline follicles are missing in Fig. C), but the hooks are identical with those of *G. bengalensis* (Fig. A) and also other morphological characteristics correspond to those of the latter species. Consequently, *G. seenghali* becomes a new synonym of *G. bengalensis*.

Hiware and Jadhav [27] described *G*. *maharashtrii* from *W*. *attu* from Satara, Maharashtra, India. Later, researchers from the same laboratory in Aurangabad proposed additional three species found in the same host (*W*. *attu*) and locality, namely *G*. *dharurensis* Jadhav and Tat, 1997; *G*. *ramkaei* Pawar and Hiware, 2008; and *G*. *bendsurensis* Reddy, Wankhede, Dhole and Anand, 2011. The validity of these taxa is questionable because the authors studied contracted and digested specimens (see Figs. A–D of Hiware and Jadhav [27]; Figs. A and B in Jadhav and Tat [28]; very low-quality photomicrographs of Reddy et al. [39]). Moreover, illustrations are very schematic and undoubtedly do not document the actual morphology of the worms, as obvious from various incorrect structures (see figures of Hiware and Jadhav [27]; Jadhav and Tat [28]; Pawar and Hiware [37]; Reddy et al. [39]), such as the shape of hooks, erroneous morphology of the cirrus-sac and genital atrium, extent and position of vitelline follicles, and shape of the eggs (Pawar and Hiware [37] reported the eggs to be elongate, resembling those of caryophyllidean or bothriocephalidean cestodes, whereas all species of *Gangesia* have spherical eggs – [3], [63]).

The morphological descriptions mentioned above also contain apparent mistakes, such as the absence of the neck in Hiware and Jadhav [27] and the diameter of the suckers larger than the width of the scolex [sic!] in Reddy et al. [39]. Discrepancies also exist between the text and figures: the number of hooks was reported in the text to be 40–45, but only 35 hooks were illustrated in Fig. A of Hiware and Jadhav [27], the number of the testes mentioned in the text (60–70) markedly differs from that illustrated by Jadhav and Tat [28] in Fig. B (32 testes only). As a rule, type specimens do not exist, as confirmed on site (Dr. Babasahed Ambedkar Marathwada University, Aurangabad, Maharashtra) by one of the present authors (M.O.). Since all four species mentioned above, i.e. *G*. *bendsurensis*, *G*. *dharurensis*, *G*. *maharashtrii* and *G*. *ramkaei*, are almost indistinguishable from *G*. *bengalensis* (see Table 1), they are synonymized with it unless new data based on well-fixed material provide unequivocal evidence of their validity.

*Gangesia cirrhinae* was described by Patel et al. [30] from the cyprinid fish *Cirrhina mrigala* ( = *Cirrhinus cirrhosus*) from Maharashtra, India. The original description was based on decomposed specimens (see Fig. 1A, B of Patel et al. [30]) and morphological description was very poor and erroneous, e.g., hooks on the rostellum-like organ were described to form as many as 5 rows (but only 27 hooks were reported to be present, which would imply that each row contains only 5–6 hooks, which is non-sense), hooklets on the suckers were not reported (they are in fact present in all species of *Gangesia* – [1], [2], [56], [57], [63]), no cross sections were provided and the number of uterine diverticula was not reported. However, it is obvious from Fig. 1B of Patel et al. [30] that the hooks are arranged in two rows, which is a typical feature of *G. bengalensis*. Even though conspecificity of both species cannot be confirmed (type specimens were never deposited – B. Jadhav, pers. comm.), *G*. *cirrhinae* is synonymized with *G*. *bengalensis*.

Shinde et al. [31] very briefly described *G*. *rohitae* from *Labeo rohita* from Maharashtra, India. It seems that the description was based on immature specimens (the uterus was not yet developed – see Fig. B in Shinde et al. [31]) and it is apparently erroneous in several characteristics (e.g., the number of hooks was mentioned to be 60 in the text whereas only 44 hooks, i.e. 25 in the upper row and 19 in the lower row, were illustrated – Fig. A). Based on the presence of two rows of hooks of similar length and other morphological characters (see Table 1), *G*. *rohitae* is considered to be conspecific with *G*. *bengalensis*.

In 2004, G.B. Shinde with co-authors [34] described another new species from the same host (*L*. *rohita*) and locality in Maharashtra and proposed the identical name [sic!], i.e. *Gangesia rohitae*. This implies that *Gangesia rohitae* Pawar, Lakhe, Shinde and Patil, 2004 becomes a homonym of *G*. *rohitae* Shinde, Mahajan and Begum, 1999, the latter taxon being a synonym of *G. bengalensis*.

Bhure et al. [40] reviewed the species of *Gangesia* from freshwater fishes from Maharashtra, India, and described another new species, *G*. *marathwadaensis* from *W*. *attu*. The description is very short (no measurements of any structure are given), erroneous (description of *G*. *mastacembali* Wankhede, 2005 is provided instead of that of *G*. *marathwadaensis*) and supplemented with very schematic and uninformative line drawings (see Figs. 2Q and 3Q in [40]). The most characteristic feature of this species, i.e. the number of hooks, corresponds to that of *G*. *bengalensis*, with which it is now synonymized.

Bhavare and Shukla [41] described a new species *G*. *jayakwadensis* from walking catfish *Clarias batrachus* from Aurangabad, India, but the description is brief, with several errors and illustrations are very schematic. However, the diagnostic characteristics such as a double circle of rostellar hooks and their number (see Table 1) correspond to those of *G*. *bengalensis*, with which it is synonymized.

Chavan [64] proposed two new species, *G*. *attui* and *G*. *parbhaniensis*, from *W*. *attu*, both being indistinguishable from *G*. *bengalensis* (see Plates iv and ii of Chavan [64]). However, descriptions of both species were presented in an unpublished thesis and thus *G*. *attui* and *G*. *parbhaniensis* become *nomina nuda*.

**Figure 4:**
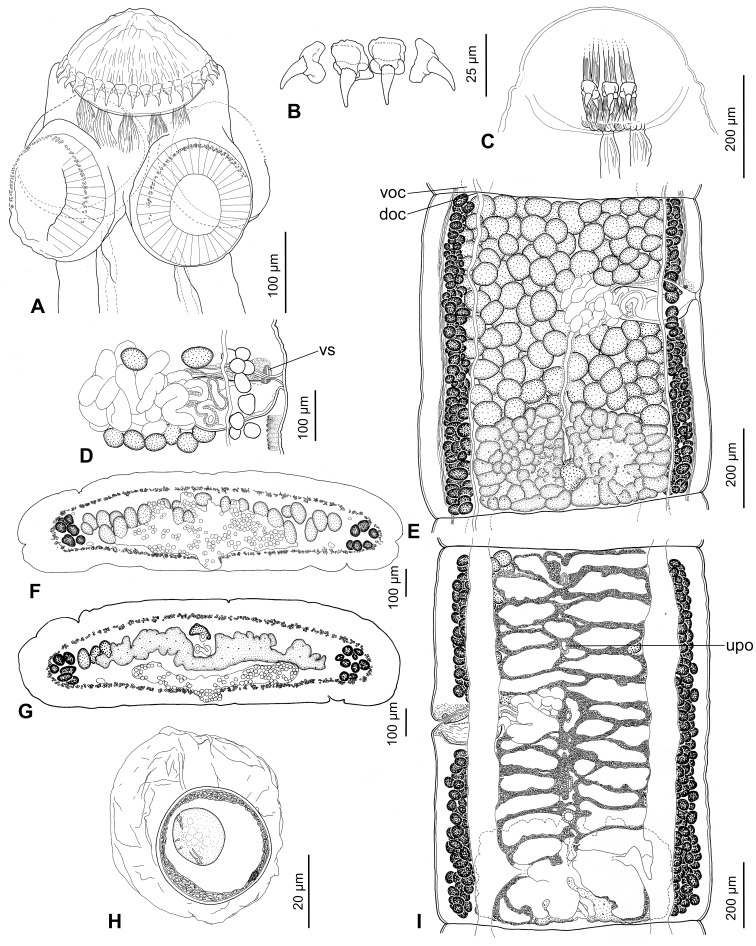
*Gangesia agraensis* Verma, 1928 from *Wallago attu*, India. A. Scolex, dorsoventral view (IPCAS C-617, field no. IND 167). **B.** Rostellar hooks (MHNG-PLAT 82298, field no. IND 795B). **C.** Detail of retractor muscles. **D.** Terminal genitalia, dorsal view (IPCAS C-617, field no. IND 795). **E.** Mature proglottis, dorsal view (IPCAS C-617, field no. IND 167). **F**, **G.** Cross sections at level of testicular field and ovary, respectively (MHNG-PLAT 60725, field no. 117/08). **H.** Egg drawn in distilled water. **I.** Gravid proglottis, ventral view (IPCAS C-617, field no. IND 795). Abbreviations: **doc** – dorsal osmoregulatory canal, **upo** – uterine pore- like opening, **voc** – ventral osmoregulatory canal, **vs** – vaginal sphincter.

**Figure 5:**
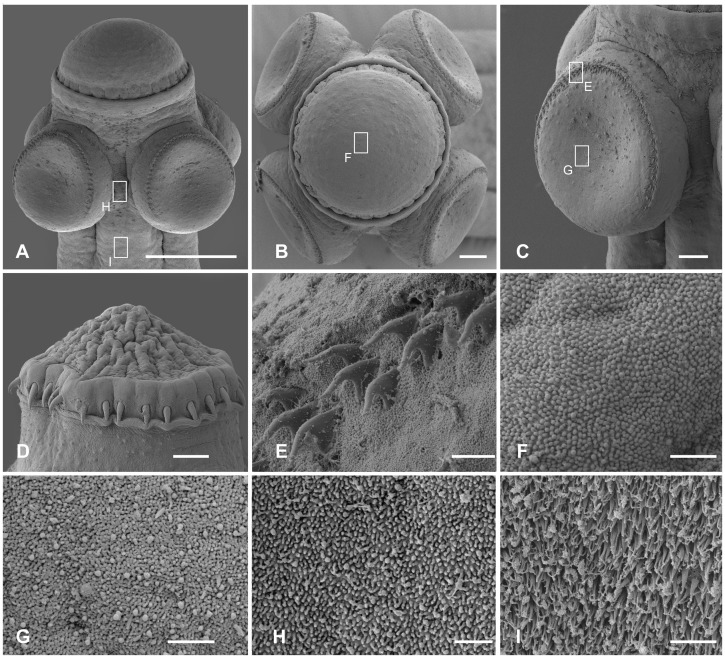
Scanning electron photomicrographs of the scolex of *Gangesia agraensis* Verma, 1928. **A.** Scolex, dorsoventral view. **B.** Scolex, apical view. **C.** Sucker with small hooklets on the outer rim. **D.** Detail of rostellum-like organ, dorsoventral view; note one row of rostellar hooks. **E.** Detail of the outer rim of the sucker with hooklets. **F.** Detail of papilliform filitriches on the rostellum-like organ. **G.** Detail of papilliform filitriches on the sucker. **H.** Detail of acicular filitriches in between the suckers. **I.** Detail of capilliform filitriches and small gladiate spinitriches on the neck region. Scale bars: **A** –100 µm; **B**–**D** –20 µm; **E**, **I** –2 µm; **F**–**H** –1 µm.

Fernando and Furtado [65] reported *G*. *bengalensis* from *W*. *attu* from northern Ceylon (Sri Lanka), but they in fact found two species, *G*. *bengalensis* and *G*. *agraensis*, as obvious from their morphological descriptions and illustrations (Figs. 1g and 1h in Fernando and Furtado [65]). Gupta and Arora [14] misidentified specimens of *G*. *bengalensis* from *W*. *attu* as *G*. *lucknowia* and *G*. *macrones*, as obvious from comparison of their morphology, including the armature of the scolex (see Figs. 4, 5, 15 and 16 of Gupta and Arora [14]).

#### Gangesia agraensis Verma, 1928

Synonyms: *Gangesia wallago* Woodland, 1924 (*in part*); *G*. *jammuensis* Fotedar and Dhar, 1974 (*nomen nudum*); new synonyms and invalid names: *G*. *haryanae* Gupta and Arora, 1979; *G*. *sanehensis* Malhotra, Capoor and Shinde, 1981; *G*. *shindei* Deshmukh and Shinde, 1989; *G*. *wallagoi* Chavan, 1997 (*nomen nudum*); *G*. *clariusae* Jadhav, Budrukkar, Babare, Bangale and Pawar, 2001; *G*. *ambikaei* Hiware, Kakade and Reddy, 2004; *G*. *batrachusi* Begum, 2007; *Silurotaenia nybelini* Shinde, Deshmukh and Chincholikar, 1975.

Redescription (based on 27 specimens from *W*. *attu* from Cambodia and India including 4 scoleces observed using SEM): Strobila with acraspedote proglottides, more than 81 mm long (100 mm or more according to Verma, 1928 [2]) and up to 950 wide (1.4 mm according to Verma, 1928 [2]). Proliferative zone 5.2–8.0 mm long and 280–315 wide (n = 6). Strobila consists of more than 200 proglottides (n = 3): more than 150 immature, only 3–4 mature, 5–10 pregravid and gravid (actual number is higher because posteriormost proglottides were cut for molecular analyses). Proglottides variable in shape, mature proglottides usually longer (0.81–1.04 mm) than wide (695–950; n = 8).

Scolex wider than neck, 270–300 long by 305–340 wide (n = 8). Rostellum-like organ 125–135 long and 150–160 wide, armed with single row of uniform hooks. Rostellar hooks 28–32 in number, 24–27 long (n = 20; blade 12–16); basal plate almost rectangular, 13–16 wide, with shallow constriction in centre (see Fig. 4B). Suckers four, uniloculate, 140–175 in diameter (n = 20); R/S = 0.85–1.06. Outer rim of anterior 1/2–1/3 of suckers covered with minute (2–3 long) hamulate-shaped hooklets, *c*. 100 in total (n = 3), arranged in three, rarely two rows (n = 28; Figs. 4A; 5A,C,E).

Strong retractor muscle bundles surround rostellum-like organ, joined each other at level of suckers and forming wide muscle band at neck region (Figs. 4A,C; 9A).

Rostellum-like organ and suckers uniformly covered with papilliform filitriches (Fig. 5F,G). Acicular filitriches present between suckers (Fig. 5H). Neck region covered with small gladiate spinitriches, smaller than those on neck of *G. bengalensis* (Fig. 5I).

Inner longitudinal musculature well developed, forming isolated bundles of muscle fibres. Subtegumental muscles well developed. Ventral osmoregulatory canals very wide (70–210; n = 8), which represents 16–22% of width of mature proglottis, thin-walled, overlapping lateralmost testes, may form transverse commissures in some proglottides. Dorsal osmoregulatory canal narrow, thick-walled, may reach vitelline follicles laterally (Fig. 4D,E).

Testes medullary, in one field, spherical to oval, 50–70 in diameter (n = 40), numbering 140–170 (n = 8), mostly forming two or three incomplete layers (Fig. 4E,F), occupying 3/4 of proglottis length. Cirrus-sac elongate, thick-walled, 175–225 long, 90–110 wide (L/W ratio = 1.7–2.4; n = 15); C/P = 20–39% (1/5–2/5). Genital pores irregularly alternating, pre-equatorial to equatorial, situated at 33–53% (n = 15) of length of mature proglottides from anterior margin.

Ovary medullary, bilobed, 490–515 wide (n = 8), each lobe almost rectangular, with numerous lobules extending both dorsally and ventrally; ovary 245–365 long, occupying *c.* 1/3 (32–37%) of length of mature proglottides (Fig. 4E). O/P = 68–72%. Mehlis’ gland 80–100 in diameter (n = 8), representing 8–10% of proglottis width. Vagina thick-walled, posterior (61%; n = 85) or anterior (39%) to cirrus-sac, with higher concentration of chromophilic cells in its distal (terminal) part, and ring-like vaginal sphincter near genital atrium (Fig. 4D).

Vitelline follicles medullary, in two longitudinal bands on both sides of proglottis, occupying almost its total length in mature proglottides (96–99% on poral side, 95–99% on aporal side; n = 8); bands interrupted at level of terminal genitalia on ventral side, with few follicles on dorsal side (Fig. 4D).

Uterus medullary, with development of type 1 according to de Chambrier et al. [55], defined as follows: in immature proglottides, uterine stem present as longitudinal concentration of chromophilic cells along median line. Lumen of uterus appears in last premature proglottides, gradually extending to form thick-walled tubular structure. Eggs appear simultaneously with formation of lateral, thin-walled diverticula. In pregravid proglottides, lateral diverticula remain thin-walled, encircled by thick chromophilic cell layer, 14–20 in number on each side (n = 20), occupy up to 63% of proglottis width (Fig. 4I), may completely overlap ovary (Fig. 4I). Uterus opened with 5–8 pore-like openings (Fig. 4I).

Eggs with hyaline, spherical outer envelope, 55–65 (measured in eggs from uterus in distilled water; n = 25) in diameter; embryophore thick (2–3 wide), spherical, 30–35 in diameter, consisting of two layers; outer layer thinner than nuclei-containing envelope; oncosphere spherical, 15–20 in diameter, with three pairs of embryonic hooks, each 6–7 (n = 10) long (Fig. 4H).

*Type-host*: *Wallago attu* (Bloch and Schneider, 1801) (Siluriformes: Siluridae).

*Other hosts*: *Cirrhinus cirrhosus* (Bloch, 1795), *Puntius ticto* (Hamilton, 1822) (Cypriniformes: Cyprinidae); *Proeutropiichthys taakree* (Sykes, 1839) (Siluriformes: Schilbeidae); *Clarias batrachus* (Linnaeus, 1758) (Siluriformes: Clariidae).

*Type-locality*: Agra, Uttar Pradesh, India.

*Material studied*: 16 specimens from *W. attu* from the Atreyee River in Balurghat (AA 28–25. x. 2007; IND 158, 165, 166, 167–5. iii. 2009), the Hooghly River in Rishra (AA 86; 24. ii. 2008), all West Bengal, India, collected by A.A., T.S. and P.K.K. (BMNH 2012.8.23.1–3; IPCAS C-617; MHNG-PLAT 63241, 63250, 82297–82300, 82310; USNPC 105945–105947; ZSI – not accessioned); three specimens from *W. attu* from the Brahmaputra River in Guwahati, Assam, India (IND 795 and 814–17. and 18. iii. 2011), collected by A.A., T.S. and M.O.; four specimens from *W. attu* from fish market in Vaijapur, Maharashtra, India (117/08– on 18. iii. 2008), collected by M.O. (MHNG-PLAT 60725); six specimens from *W. attu* from the Bhami River in Siddheshwari, Maharashtra, India (MH 6–3. ix. 2010), collected by S.P.C. (IPCAS C-617; MHNG-PLAT 82299); and one specimen from *W. attu* from fish market in Phnom Penh, Cambodia (VNT 431– on 18. x. 2010), collected by A.C. and T.S. (MHNG INVE 75452).

*Site of infection*: intestine.

*Prevalence*: 63% (n = 8) in Balurghat, West Bengal and 20% in Rishra, West Bengal (n = 5). 50% (n = 4) in Vaijapur, Maharashtra.

*Intensity of infection*: 1–9 tapeworms (mean 3) in Balurghat, West Bengal and 1–3 tapeworms (mean 2) in Aurangabad, Maharashtra.

*Distribution*: Cambodia, India (Assam, Haryana, Karnataka, Kashmir, Maharashtra, Uttar Pradesh, West Bengal) and Sri Lanka.

*Remarks*: The species was described by Verma [2], who first realized that two species of *Gangesia*, differing from one another by the number of rows (circles) of hooks on the rostellum-like organ and other morphological characteristics, may occur simultaneously in *Wallago attu*. Verma [2] provided a reasonably good morphological description of *G. agraensis*, but his specimens were contracted and strongly flattened (see his Figs. 38–40 and 43–48). However, Southwell [5] synonymised *G*. *agraensis* with *G*. *bengalensis*, which was followed by most subsequent authors (see below and remarks on *G*. *bengalensis*).

The present study provides unequivocal support of the validity of *G. agraensis* based on morphological and molecular data and thus fully confirms Verma’s [2] conclusions. The present study also provides new information on the morphology of *G. agraensis*: (i) the anterior half of the outer rim of the sucker is covered by 2–3 rows of small hamulate shaped hooklets, whereas Verma [2] reported only one, occasionally two rows of minute spines; (ii) ventral osmoregulatory canals are markedly wide, occupying 16–22% of the width of mature proglottides (wide canals were observed in all specimens, which were fixed in the same way as those of *G. bengalensis*, in which ventral canals are always much narrower; this implies that the canals in *G. agraensis* were not inflated during fixation); (iii) base of hooks on the rostellum-like organ is almost rectangular in shape with shallow constriction in the centre; (iv) vitelline follicles are exclusively in the medulla, unlike those of *G*. *bengalensis*, *G*. *macrones* and *G*. *vachai*, in which some follicles are paramuscular; (v) suckers are uniformly covered with papilliform filitriches.

Since Verma’s [2] comprehensive account, a number of new species have been described from the Indian subcontinent, especially from Maharashtra. Unfortunately, all these descriptions were incomplete and/or erroneous and were always based on deformed (strongly compressed) or macerated (with detached hooks and disintegrated tissues) specimens. Type material of none of these species, which seem to be indistinguishable from the Verma’s taxon, is available and it is not known whether it actually exists (any request for specimens to the authors of individual taxa has never been replied).

Based on comparison of the original and present data on *G. agraensis* with those provided in the original descriptions of Indian authors ([14], [16], [23], [27], [32], [33], [36], [37]), the following species are newly synonymized with *G. agraensis* (see Table 2) and brief comments on them are provided.

*G*. *haryanae* was described by Gupta and Arora [14] from *W*. *attu* from Ambala, India. Diagnostic features of *G*. *haryanae* are irregularly arranged 2–20 [sic!] hooks on the rostellum-like organ and suckers without hooklets, but it is evident that the specimens were partially digested and hooks and hooklets on the suckers were lost due to tissue decomposition (only six hooks irregularly scattered on the scolex are present in Fig. 1 of Gupta and Arora [14]). The description was very brief and incomplete (e.g., the information on uterine branches is missing). Except for the absence of hooklets on the suckers, which is undoubtedly a result of disintegration of the tegument, *G*. *haryanae* possesses all characteristics that are typical of *G*. *agraensis*. Therefore, both taxa are considered to be conspecific and *G. haryanae* becomes a junior synonym of *G. agraensis*.

Malhotra et al. [16] described *G*. *sanehensis* from the cyprinid *Cirrhina mrigala* ( = *Cirrhinus cirrhosus*) and from *W*. *attu* from Garhwal ( = Uttarakhand), India. Morphological description appears to be detailed but illustrations are very schematic and do not provide sufficient evidence about reliability of morphological data. For example, hooks on the rostellum-like organ were not illustrated in detail to confirm their shape and unusually high size variability, which was not observed in any other species. The illustration of a mature proglottis (Fig. 3 of Malhotra et al. [16]) was insufficient because it did not provide accurate information on the distribution and number of testes, internal structure of the cirrus-sac, position (extent) of vas deferens and other features. Vitelline follicles in a gravid proglottis (Fig. 4 of Malhotra et al. [16]) were not correctly illustrated and the ovary was missing. Despite these obvious mistakes and deficiencies in the morphological description, it is unquestionable that *G*. *sanehensis* shares key diagnostic characteristics with *G*. *agraensis*, i.e. a single row of hooks of identical shape, suckers armed with a few rows of hooklets, a large, massive strobila, and a similar number of testes (see Table 2). Consequently, *G*. *sanehensis* is synonymized with *G*. *agraensis*.

*Gangesia shindei* was described by Deshmukh and Shinde [23] from the barb *Barbus ticto* ( = *Puntius ticto*) from Aurangabad, India and the species was named after one of the authors themselves (!). Species description was very brief and incomplete (e.g., there are no data on uterine branches and eggs). It is also evident from the very schematic figures that the figured specimen was partially decomposed and extremely flattened (see Fig. 1 of Deshmukh and Shinde [23]). However, the characteristics considered to be species-specific (see above – Species composition and differential criteria), such as a single row of hooks on the rostellum-like organ, their number and size, and the number of testes, are fully consistent with those of *G*. *agraensis* (see Table 2). Thus *G*. *shindei* is synonymized with *G*. *agraensis*.

Jadhav et al. [32] described *G*. *clariusae* from the walking catfish, *Clarias batrachus*, from Karnataka, India, and Begum [36], who worked in the same laboratory (Aurangabad), described *G*. *batrachusi* from the same host and locality. The descriptions of both taxa were based on decomposed specimens and were very brief and erroneous (e.g., subtegumental cells were misinterpreted as vitelline follicles, see Fig. C of Jadhav et al. [32] and Fig. 1C of Begum [36]). Figs. 1A and 1B in Begum [36] and Fig. A in Jadhav et al. [32] clearly show that only one row of hooks is present in both species and that their base is rectangular (see Fig. B of Jadhav et al. [32] and Fig. 1B of Begum [36]), which fully corresponds to the morphology of *G. agraensis* (see Table 2). Based on this morphological similarity, both the taxa are synonymised with *G*. *agraensis*.

Hiware et al. [33] described *G*. *ambikaei* from *W*. *attu* from Maharashtra, India. Description was based on material of very poor quality as obvious from the illustrations provided (see Figs. A–C of Hiware et al. [33]). Hooks were illustrated very schematically (Fig. B of Hiware et al. [33]) and the uterine stem was apparently incomplete; vitelline follicles and vas deferens were almost completely missing (Fig. C of Hiware et al. [33]). Due to poor quality of the material and incomplete morphological description, it is difficult to rely on the data provided (type specimens do not exist – B. Jadhav, pers. comm.). In fact, some measurements were apparently erroneous, as obvious from discrepancies between the text and figures (some testes are illustrated as postovarian in Fig. C of Hiware et al. [33], which is apparently error because no species of *Gangesia* has testes posterior to the ovary). The position of the hooks indicates that only one row was present, but their number was reported to be slightly higher than that of *G*. *agraensis*. Despite this negligible difference, both species are considered conspecific and *G*. *ambikaei* becomes a new synonym of *G*. *agraensis*.

Fotedar and Dhar [12] described *G*. *jammuensis* from *W*. *attu* from Jammu and Kashmir, India. This species is in fact *G*. *agraensis*, and was already invalidated by Ash et al. [66]. Chavan [64] proposed a new species, *G*. *wallagoi*, which is indistinguishable from *G*. *agraensis* (see Plate III of Chavan [64]). However, description of the former species has never been published and *G*. *wallagoi* should be considered as *nomen nudum*.

Gupta and Arora [14] misidentified specimens of *G*. *agraensis* from *W*. *attu* as *G*. *parasiluri*, as obvious from comparison of their morphology, including armature of the scolex (see Figs. 10–14 of Gupta and Arora [14]). *Gangesia parasiluri* is a specific parasite of the Amur catfish *Silurus asotus* in Japan and Russian Far East ([3], [67], [68], [69]) and differs in its morphology from *G*. *agraensis*; both taxa are also not closely related (see Fig. 10).

Shinde et al. [13] described *Silurotaenia nybelini* from *Pseudeutropius taakree* ( = *Proeutropiichthys taakree*) from Maharashtra, India, but there was conspicuous discrepancy between the text and figures: the text claims the presence of minute hooks on the rostellar-like organ, but they were not illustrated – see Fig. A in Shinde et al. [13]. There were also no traces of hooklets on the suckers. It seems that the authors observed *Gangesia* specimens devoid of hooks and hooklets due to their *post mortem* decomposition. They also provided erroneous data, e.g., measurements of the rostellum-like organ (470 µm×720 µm) are greater than the diameter of the whole scolex (140 µm×100 µm) and the suckers are much bigger in Fig. A than those described in the text. *Silurotaenia nybelini* possesses significantly wide ventral osmoregulatory canals (see Figs. B, C of Shinde et al. [13]) and also other characteristics correspond to those typical of *G*. *agraensis* (see Table 2). Based on this similarity, *S. nybelini* is considered to be a synonym of *G. agraensis*.

#### Gangesia macrones Woodland, 1924

Synonyms (all new): *Silurotaenia paithanensis* Shinde, Majid and Solunke, 1983; *S*. *barbusi* Shinde, Kadam and Jadhav, 1984; *S*. *macroni* Shinde, Kadam and Jadhav, 1984; *S*. *singhali* Shinde, Kadam and Jadhav, 1984; *S*. *behairvnathi* Deshmukh and Shinde, 1989; *S. shastri* Gavhane and Jadhav, 1991; *S*. *raoii* Bhure, Pathan, Nanware and Dhondge, 2010; *Gangesia mastacembali* Wankhede, 2005.

Redescription (based on ten specimens collected from *S*. *seenghala* from Bangladesh and India, including three scoleces observed in SEM): Strobila with acraspedote proglottides, more than 65 mm long (28–56 mm according to Woodland, 1924 [1]) and up to 1.15 mm wide (a little over 1 mm according to Woodland, 1924 [1]). Proliferative zone 3.6–4.3 mm long and 165–195 wide (n = 4). Strobila consists of more than 110 proglottides (n = 3): 80–86 immature, only 3–4 mature, 10–20 pregravid and gravid (actual number is higher because posteriormost proglottides were cut for molecular analyses). Proglottides variable in shape, mature proglottides usually wider (0.96–1.16 mm) than long (0.63–1.10 mm; n = 8), but gravid proglottides longer (1.4–3.1 mm) than wide (0.73–1.2 mm; n = 15).

**Figure 6:**
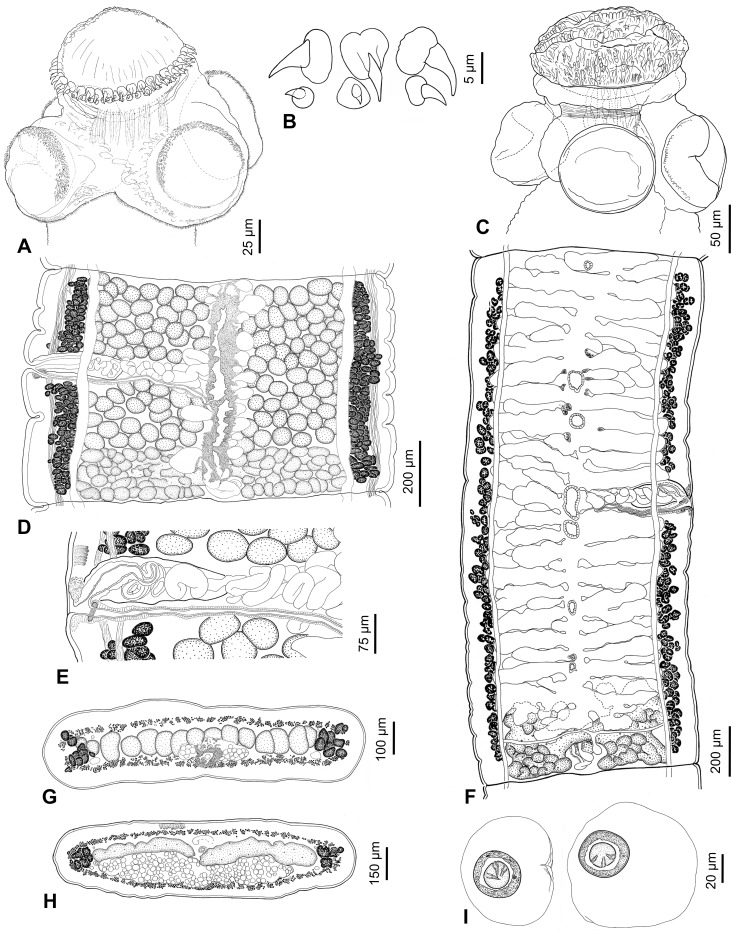
*Gangesia macrones* Woodland, 1924 from *Sperata seenghala*, India. A. Scolex, dorsoventral view (IPCAS C-618, field no. MS 4h). **B.** Rostellar hooks (IPCAS C-618, field no. MS 4h). **C.** Scolex of decomposed specimen fixed with cold formalin and detached hooks and hooklets (IPCAS C-618, field no. MH 26). **D.** Mature proglottis, ventral view (MHNG-PLAT 82302, field no. MS 6r). **E.** Terminal genitalia, ventral view (IPCAS C-618, MS 4h). **F.** Gravid proglottis, ventral view (IPCAS C-618, field no. MS 4h). **G**, **H.** Cross sections at level of testicular field and ovary, respectively (IPCAS C-618, field no. MS 4k). **I.** Eggs drawn in distilled water.

**Figure 7:**
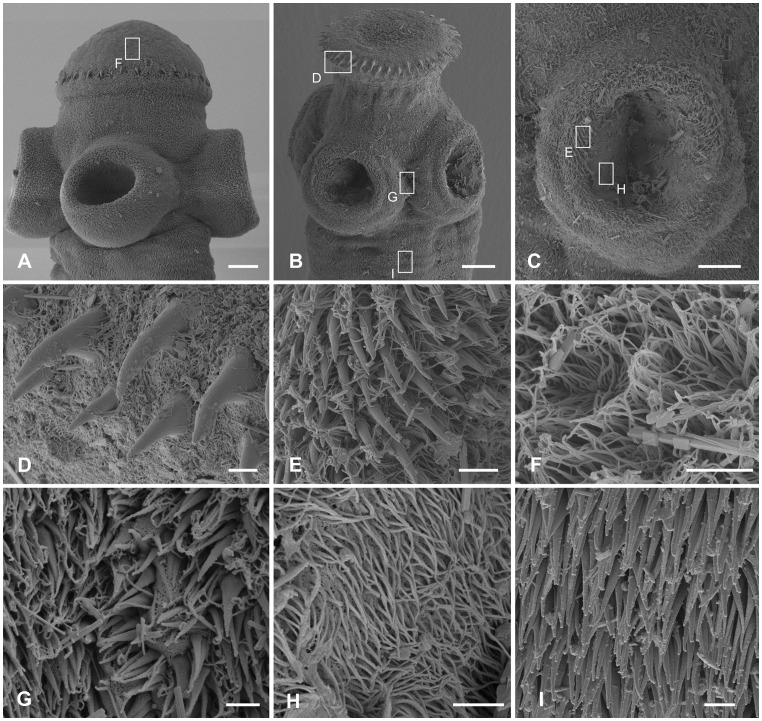
Scanning electron photomicrographs of the scolex of *Gangesia macrones* Woodland, 1924. **A**, **B.** Scolex, dorsoventral view. **C.** Sucker with small hooklets on the outer rim. **D.** Detail of a rostellum-like organ; note two rows of rostellar hooks of different shape with capilliform filitriches. **E.** Detail of the outer rim of the sucker with hooklets and capilliform filitriches. **F.** Detail of capilliform filitriches on the rostellum-like organ. **G.** Detail of capilliform filitriches and gladiate spinitriches in between the suckers. **H.** Detail of capilliform filitriches on the sucker. **I.** Gladiate spinitriches on the neck region. Scale bars: **A**, **B** –30 µm; **C** –20 µm; **D**–**F** –2 µm; **G**–**I** –1 µm.

Scolex wider than neck, 160–220 long by 215–275 wide (n = 5). Rostellum-like organ 90–115 long and 110–175 wide, armed with double row of hooks. Rostellar hooks usually alternating with each other, 80–85 in total (upper row includes 38–40 hooks; lower row 42–45 hooks), different in length. Hooks in upper row 10–13 long (n = 20; blade 6–8 and widely oval basal plate 8–10 long), and those in lower row 5–7 long (n = 20; blade 2–4 long and spherical basal plate 5–7 in diameter) (Fig. 6B). Suckers four, uniloculate, 80–90 in diameter (n = 18); R/S = 1.27–1.95. Outer rim of anterior 1/2–1/3 of suckers covered with numerous minute (1–2 long) hooklets, arranged in about 9–12 (n = 6; precise number hard to count) rows (Figs. 6A; 7B,C).

Strong retractor muscle bundles surround rostellum-like organ, joined each other at level of suckers, forming wide muscle band at neck region (Figs. 6A; 9C,H).

Rostellum-like organ and suckers uniformly covered with capilliform filitriches (Fig. 7E,F,H). Both capilliform filitriches and gladiate spinitriches interspersed between suckers (Fig. 7G). Neck region covered with gladiate spinitriches (Fig. 7I).

Inner longitudinal musculature well developed, anastomosed, forming isolated bundles of muscle fibres. Subtegumental muscles well developed. Ventral osmoregulatory canals thin-walled, narrow (their width represents 3–4% of width of mature proglottis), overlapping lateralmost testes, may form transverse commissures in some proglottides. Dorsal osmoregulatory canal narrow, thick-walled, may reach vitelline follicles laterally.

Testes medullary, in one field, spherical to oval, 55–70 in diameter (n = 40), numbering 90–140 (n = 8), forming single layer (Fig. 6D,G), occupying 3/4 of proglottis length. Cirrus-sac elongate, thick-walled, 255–290 long by 75–85 wide (n = 15; L/W ratio = 3.1–3.7); C/P = 22–28%. Genital pore irregularly alternating, almost equatorial, situated at 42–46% (n = 15) of proglottis length from anterior margin.

Ovary medullary, bilobed, 760–860 wide (n = 8), each lobe almost oval (in mature proglottides) with numerous lobules extending both dorsally and ventrally; ovary 145–225 long (Fig. 6D), occupying 1/5–1/4 (22–25%) of length of mature proglottides (Fig. 6D), O/P = 73–83%. Mehlis’ gland 80–100 in diameter (n = 8), representing 8–11% of proglottis width. Vagina thick-walled, posterior (64%; n = 80) or anterior (36%) to cirrus-sac, with higher concentration of chromophilic cells in its distal (terminal) part, and ring-like vaginal sphincter near genital atrium (Fig. 6E).

Vitelline follicles medullary, with some follicles paramuscular (penetrating between muscle fibres of inner longitudinal musculature), in two longitudinal bands on both sides of proglottis, occupying almost its total length (94–95% on poral side, 93–95% on aporal side in mature proglottides; n = 8); bands interrupted at level of terminal genitalia on ventral side, with few follicles on dorsal side (Fig. 6E).

Uterus medullary, with development of type 1 according to de Chambrier et al. [55], defined as follows: in immature proglottides, uterine stem present as longitudinal concentration of chromophilic cells alongside median line. Lumen of uterus appears in last premature proglottides, gradually extending to form tubular structure. Eggs appear simultaneously with formation of lateral, thin-walled diverticula. In pregravid proglottides, lateral diverticula remain thin-walled, 25–35 in number on each side (n = 25), occupy up to 83% of proglottis width (Fig. 6F), may partially overlap ovary (Fig. 6F). Uterus opened with 7–10 pore-like openings (Fig. 6F).

Eggs with hyaline, spherical outer envelope, 65–85 in diameter (measured in eggs from uterus in distilled water; n = 30); embryophore thick (3–4 wide), spherical, 28–30 in diameter, consisting of two layers; outer layer thinner than nuclei-containing envelope; oncosphere spherical, 18–20 in diameter, with three pairs of embryonic hooks, 8–10 long (Fig. 6I).

*Type-host*: *Sperata seenghala* (Sykes, 1839) (Siluriformes: Bagridae).

*Other hosts*: *Puntius ticto* (Hamilton, 1822) (Cypriniformes: Cyprinidae); *Mastacembelus armatus* (Lacépède, 1800) (Synbranchiformes: Mastacembelidae).

*Type-locality*: Allahabad, Uttar Pradesh, India.

*Material studied*: Nine specimens from *S. seenghala* from Yeldari dam lake on the Purna River, Maharashtra, India (MH 4, 6, 18–20, 22–24, 32, 39–16. i., 6. iii., 20. viii. and 20. x. 2010, and 12. ii. 2011), collected by S.P.C. (BMNH 2012.8.23.6–7; IPCAS C-618; MHNG-PLAT 77755, 82301–82304; USNPC 105948–105949; ZSI – not accessioned), one specimen from *S. seenghala* from the Old Brahmaputra River in Mymensingh, Bangladesh (BAN 61–4. iii. 2011), collected by A.A., T.S and M.O. (IPCAS C-618).

*Site of infection*: intestine.

*Prevalence*: 25% (n = 20) in Nanded, Maharashtra, India in 2012.

*Intensity of infection*: 1–7 tapeworms (mean 2.4) in Nanded, Maharashtra, India.

*Distribution*: Bangladesh and India (Maharashtra, Uttar Pradesh).

*Remarks*: Woodland [1] described *G*. *macrones* from seven or eight flattened specimens (but only with two scoleces) from the giant river catfish *Sperata seenghala*. The original description was not complete (e.g., no data on eggs were provided) and contained some errors (e.g., the number of hooks reported in the text was 33 but as many as 47 hooks were illustrated in Fig. 26 of Woodland [1]). Material of *G. macrones* recently collected in Maharashtra, India enabled us to provide new morphological data on this cestode, which is typified by a number of morphological characteristics: (i) rostellum bears a double circle of hooks: the upper row with 38–40 larger hooks (10–13 µm long) and the lower row with 42–45 smaller hooks (5–7 µm long); (ii) the outer rim of the anterior half of the suckers is covered by minute (length 1–2 µm) hooklets arranged roughly in 9–12 rows (Figs. 6A; 7C); (iii) rostellum-like organ is markedly larger (1.3–2.0x) than the suckers; (iv) scolex is comparatively small (160–220 µm long by 215–275 µm wide), similarly as suckers (80–90 µm in diameter); (v) testes (90–140 in number) form a single layer; (vi) ovarian region is comparatively short, occupying at maximum 1/4 of the length of mature proglottides; (vii) cirrus-sac is long and narrow, the length-width ratio being 3.1–3.7; (viii) suckers are covered with capilliform filitriches; (ix) vitelline follicles are medullary, with some follicles paramuscular, i.e. penetrating between the inner longitudinal musculature (Fig. 6G,H).

As many as five *Silurotaenia* spp., namely *S. paithanensis* Shinde, Majid and Solunke, 1983; *S*. *macroni* Shinde, Kadam and Jadhav, 1984; *S*. *singhali* Shinde, Kadam and Jadhav, 1984; *S. shastri* Gavhane and Jadhav, 1991; and *S*. *raoii* Bhure, Pathan, Nanware and Dhondge, 2010, have been described from *S*. *seenghala* from India, all from the same state (Maharashtra). Descriptions of these species were very brief, incomplete (e.g., cross sections necessary for confirmation of the position of internal organs were never provided, and the number of uterine branches was not reported in all but one species; see also Table 3) and often erroneous (e.g., all species were reported to possess the suckers devoid of hooklets).

It is obvious from the figures in the original descriptions that all the above-mentioned *Silurotaenia* spp. were described from decomposed specimens that were also strongly flattened and thus deformed. Some of the authors of these taxa also misinterpreted decomposed muscle fibres (see Figs. 1A and B of Shinde et al. [20]; Plates 1A and 2A of Shinde et al. [21]) or gland cells (see Fig. A of Gavhane and Jadhav [26] and Fig. 2A of Bhure et al. [38]) as hooks on the rostellum-like organ. One of the present authors (S.P.C.) fixed originally a few decomposed specimens of *G*. *macrones* from the same host (*S*. *seenghala*) and same state (Maharashtra) in cold formalin. These worms, including their scoleces, showed a similar morphology (Fig. 6C) to that of *Silurotaenia* spp. listed above. In fact, all the above-mentioned *Silurotaenia* spp. possess all characteristic features of *G*. *macrones*, such as the shape and size of the scolex, which is small and bears conspicuous suckers, a large rostellum-like organ (its diameter is markedly larger, up to twice, than the diameter of the suckers), and a relatively short ovary (ratio of its length to the length of proglottides is about 1/4 only; see Table 3). Based on this morphological similarity, *Silurotaenia macroni*, *S. paithanensis*, *S*. *raoii*, *S. shastri* and *S*. *singhali* are synonymized with *G*. *macrones*.

Two more species of *Silurotaenia* were described from Maharashtra, both from fish hosts not typical of proteocephalidean cestodes, namely *S*. *barbusi* from the barb *Barbus ticto* ( = *Puntius ticto*) (Cyprinidae) by Shinde et al. [21], and *S*. *behairvnathi* from the zig-zag eel *Mastacembelus armatus* (Mastacembelidae) by Deshmukh and Shinde [24]. Similarly as the previously described taxa, these species were described very briefly and from decomposed material (see Plates 3, 4 of Shinde et al. [21] and Figs. A–C of Deshmukh and Shinde [24]). Both descriptions suffered from many flaws and deficiencies, e.g., muscle fibres or gland cells were considered to represent hooks on the rostellum-like organ (see Plate 3A and 4B of Shinde et al. [21] and Fig. A of Deshmukh and Shinde [24]), subtegumental cells as vitelline follicles (see Plate 3B of Shinde et al. [21]) and vitelline follicles were completely missing in illustrations (Figs. B, C of Deshmukh and Shinde [24]). Moreover, measurements provided by Deshmukh and Shinde [24] were obviously erroneous, e.g., the diameter of suckers (23 µm) was lower than the diameter of testes (27–53 µm) and the hooks were longer (71 µm) than the diameter of the rostellum-like organ (45 µm×42 µm). If doubtful characteristics and measurements are not considered, *S. barbusi* and *S. behairvnathi* possess all features typical of *G*. *macrones*, such as a small scolex, large rostellum-like organ, and similar strobilar morphology (see Table 3). For this reason, *S*. *barbusi* and *S*. *behairvnathi* are synonymized with *G*. *macrones*.

Wankhede [35] described *G*. *mastacembali* from *Mastacembelus armatus* (misspelled as *Mastacembalus armatus* in the original paper) from Aurangabad, but no illustrations were provided. Bhure et al. [40] provided two figures of this species, which clearly show that the scolex is small and possesses a large apical organ (muscle fibres of the decomposed scolex were apparently considered to be hooks). Scolex morphology and low number of testes correspond to those of *G*. *macrones* and thus *G. mastacembali* is synonymized with the former species.

#### *Gangesia vachai* (Gupta and Parmar, 1988) n. comb

Synonym: *Silurotaenia vachai* Gupta and Parmar, 1988 (new synonym).

Redescription (based on five specimens from *W*. *attu* and *Mystus* spp. from Bangladesh and India; measurements from the original description [22] in parentheses): Strobila with acraspedote proglottides, more than 25 mm long and up to 660 (600–680) wide. Proliferative zone about 0.68–1.10 mm long and 130–190 wide (n = 2). Strobila consists of more than 55 proglottides: more than 41 immature, only 3–4 mature, 5–10 pregravid and gravid (actual number is higher because posteriormost proglottides were cut for molecular analyses). Proglottides variable in shape, mature proglottides 505–630 long and 555–660 wide (n = 4).

**Figure 8:**
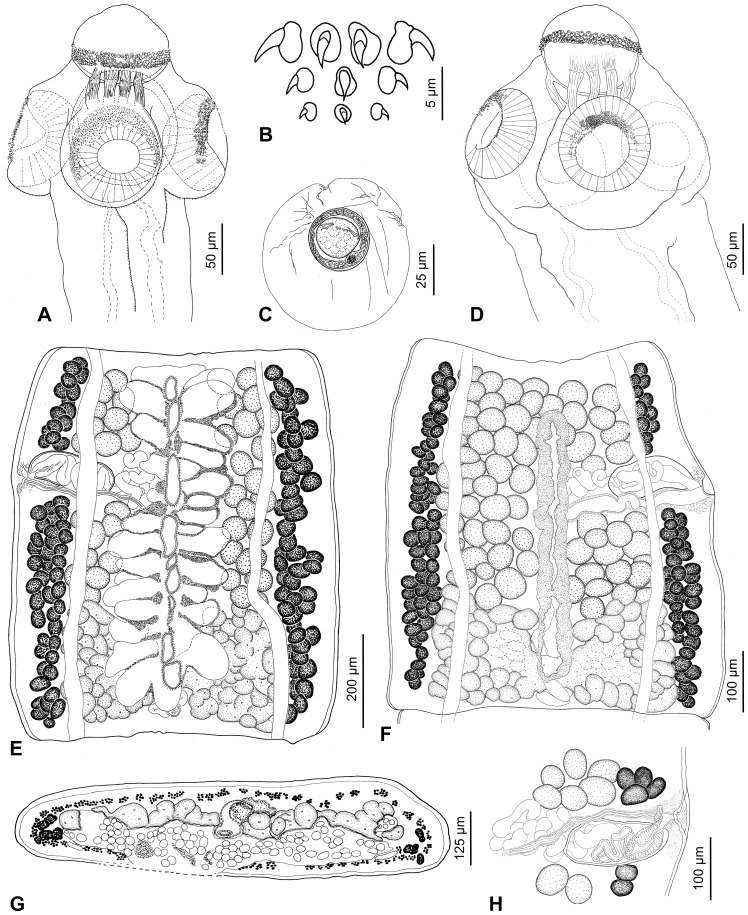
*Gangesia vachai* (Gupta and Parmar, 1988) n. comb. from *Mystus* sp., India and *Wallago attu*, Bangladesh. A. Scolex of specimen from *Wallago attu*, dorsoventral view (MHNG-PLAT 82306, field no. BAN 186). **B.** Rostellar hooks; note different sizes (distributed in 4–6 rows) (MHNG-PLAT 82306, field no. BAN 186). **C.** Egg drawn in distilled water. **D.** Scolex of specimen from *Mystus* cf. *tengara*, dorsoventral view (IPCAS C-623, field no. IND 906a). **E.** Gravid proglottis, ventral view (MHNG-PLAT 82306, field no. BAN 186). **F.** Mature proglottis, ventral view (MHNG-PLAT 82306, field no. BAN 186). **G.** Cross sections at level of the ovary (IPCAS C-623, field no. IND 906a). **H.** Terminal genitalia, ventral view (MHNG-PLAT 82306, field no. BAN 186).

Scolex wider than neck, 210–235 (349–380) long by 220–245 (320–350) (n = 2) wide. Rostellum-like organ 80–85 (140–160) long and 100 (190–220) wide. Rostellar hooks, with wide oval basal plate and short, posteriorly curved blade, arranged in 4–6 (3–5) irregular rows, ranging from 250 to 450 in total number, different in size. Size of hooks decreases from anterior (apical) row to posteriorly (Fig. 8B). Largest hooks have spherical basal plate 3–4 long and 2–3 wide, blade 3–4 long; smallest hooks with spherical basal plate 1–2 in diameter, blade 1 long (n = 30). Suckers four, uniloculate, 95–110 (130–200) in diameter (n = 8); R/S = 0.91–1.05. Outer rim of anterior 1/2–1/3 of suckers covered with numerous minute (1–2 long) hooklets, arranged in 5–11 (hard to count precisely) rows (Fig. 8A,D).

**Figure 9:**
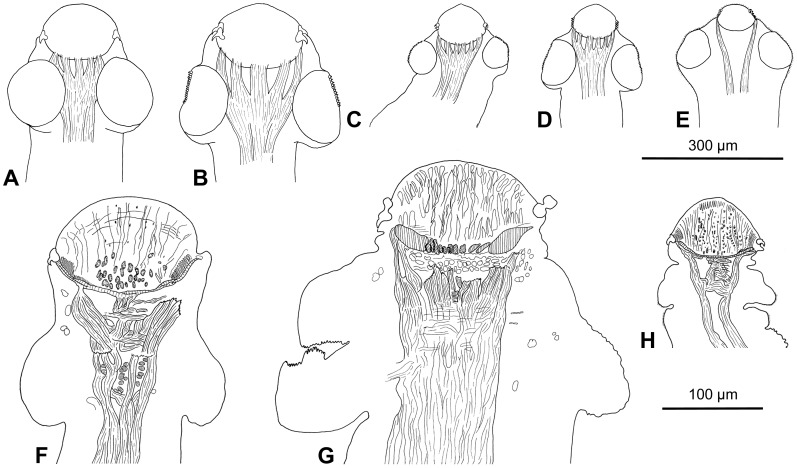
Outline of scoleces with retractor muscles of selected *Gangesia* and *Silurotaenia* taxa. A –**E.** Outline of the scoleces with retractor muscles of *Gangesia agraensis*, *G*. *bengalensis*, *G*. *macrones*, *G*. *vachai* and *Silurotaenia siluri*, respectively. **F**, **G.** Frontal section of the scoleces of *G. agraensis* (IPCAS C-617, field no. AA 86a) and *G*. *bengalensis* (IPCAS C-616, field no. AA 133), respectively. **H.** Sagittal section of the scolex of *G. macrones* (MHNG-PLAT 82303, field no. MS 22b).

Retractor muscle bundles well developed, surround rostellum-like organ, joined each other at level of suckers, form wide band at neck region (Figs. 8A,D; 9D).

Inner longitudinal musculature well developed, anastomosing, forming small isolated bundles of muscle fibres. Subtegumental muscles well developed. Ventral osmoregulatory canals thin-walled, their width representing 4–5% of width of mature proglottis (n = 4), overlapping lateralmost testes, may form transverse commissures in some proglottides. Dorsal osmoregulatory canal narrow, thick-walled, may reach vitelline follicles laterally.

Testes medullary, in one field, spherical to oval, 40–55 (n = 20; 30–50) in diameter, numbering 65–93 (100–150) (n = 4), forming single layer (Fig. 8E,F), occupying 1/3 of proglottis length. Cirrus-sac elongate, thick-walled, 135–155 (200–280) long by 50–65 (70–110) wide (n = 6; L/W ratio = 2.1–2.5); C/P = 24–25% (1/4). Genital pore irregularly alternating, pre-equatorial, situated at 54–60% (n = 6) of proglottis length from anterior margin.

Ovary medullary, bilobed, 405–510 (220–320) wide (n = 4), each lobe almost rectangular, with numerous lobules extending both dorsally and ventrally; ovary 155–200 (210–280) long (Fig. 8E,F), occupying about 1/3 (30–37%) of length of mature proglottides; O/P = 72–77%. Mehlis’ gland 65–90 in diameter (n = 4), representing 11–15% of proglottis width. Vagina thick-walled, mostly posterior (81%; n = 42) to cirrus-sac, with higher concentration of chromophilic cells in its distal (terminal) part, and ring-like vaginal sphincter near genital atrium (Fig. 8H).

Vitelline follicles medullary, with some follicles paramuscular (penetrating between muscle fibres of inner longitudinal musculature), in two longitudinal bands on both sides of proglottides, occupying almost their total length (96–97% on poral side, 94–96% on aporal side in mature proglottides); bands interrupted at level of terminal genitalia on ventral side, with few follicles on dorsal side.

Uterus medullary, with development of type 1 according to de Chambrier et al. [55]: in immature proglottides, uterine stem present as longitudinal concentration of chromophilic cells alongside median line. Lumen of uterus appears in last premature proglottides, gradually extending to form tubular structure. Eggs appear simultaneously with formation of lateral, thin-walled diverticula. In pregravid proglottides, lateral diverticula remain thin-walled, 13–18 (n = 9; 11–16) in number on each side, occupy up to 69% of proglottis width (Fig. 8E), partially overlap ovary in mature proglottides (Fig. 8E); may completely overlap ovary in gravid proglottides. Uterus with slit-like openings (Fig. 8E).

Eggs with hyaline, spherical outer envelope, 65–95 in diameter (measured in eggs from uterus in distilled water; n = 30); embryophore thick (4–7 wide), spherical, 30–35 in diameter, consisting of two layers; outer layer thinner than nuclei-containing inner envelope; oncosphere spherical, 20–25 in diameter, with three pairs of embryonic hooks, 7–10 long (Fig. 8C).

### Taxonomic Summary

*Type-host*: *Eutropiichthys vacha* (Hamilton, 1822) (Siluriformes: Schilbeidae).

*Other-hosts*: *Wallago attu* (Bloch and Schneider, 1801) (Siluriformes: Siluridae); *Mystus* cf. *cavasius* (Hamilton, 1822) and *Mystus* cf. *tengara* (Hamilton, 1822) (Siluriformes: Bagridae) (new hosts records).

*Type-locality*: Lucknow, Uttar Pradesh, India.

*Material studied*: Four specimens from *M.* cf. *cavasius* from Dhuburi, Assam (IND 786–17. iii. 2011) and *M.* cf. *tengara* from Phulbari dam lake on the Mahananda River in Siliguri, West Bengal, India (IND 303–11. iii. 2009, IND 779–16. iii. 2011, and IND 906–27. iii. 2011), collected by A.A., T.S., M.O. and P.K.K. (IPCAS C-623; MHNG-PLAT 82305), one specimen from *W. attu* from the Shomeswari River at Durgapur, Bangladesh (BAN 186–5. iii. 2011), collected by A.A., T.S. and M.O. (IPCAS C-623; MHNG-PLAT 82306).

*Site of infection*: intestine.

*Prevalence*: 5% (n = 20) in *M.* cf. *cavasius* in Assam; 5% (n = 75) in *M.* cf. *tengara* in Phulbari, West Bengal, India; 50% (n = 2) in *W*. *attu* in Durgapur, Bangladesh.

*Intensity of infection*: Always one tapeworm/fish.

*Distribution*: Bangladesh and India (Assam, Uttar Pradesh and West Bengal).

*Remarks*: Specimens recently found by the present authors are considered to be conspecific with *Silurotaenia vachai* based on their morphological similarity. This species was described by Gupta and Parmar [22] from the schilbeid catfish *Eutropiichthys vacha* from Lucknow, India.

It actually somewhat resembles *Silurotaenia siluri*, a parasite of European wels (*Silurus glanis* L.), in its morphology (see [70] and [71]). However, it is transferred to *Gangesia* as *G*. *vachai* n. comb. because it shares the following morphological characteristics with the remaining species of *Gangesia*: (1) the anterior rim of suckers is covered with several rows of small hooklets, which are markedly different from spinitriches that cover the surface of the suckers (in *S. siluri*, the suckers are covered with spinitriches only – see Figs. 1, 2 in Scholz et al. [71]); (2) the rostellum-like organ is connected with well-developed retractor muscles that form a wide band of muscles (only lateral retractor muscles are present in the scolex and neck region in *S. siluri* – see Figs. 1A–C in Scholz et al. [71] and Fig. 9E; (3) ventral osmoregulatory canals are median (internal) to vitelline follicles (versus lateral, i.e. external, to vitelline follicles in *S*. *siluri*); (4) rostellum-like organ is as large as, or slightly larger than, the suckers (versus always markedly smaller in *S*. *siluri*) [71].

**Figure 10:**
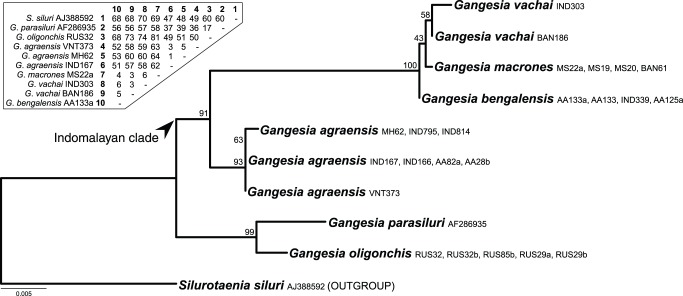
Phylogenetic analysis and lsrDNA total sequence differences of valid *Gangesia* taxa of the Indomalayan region along with all Palaearctic taxa available. Note that individual OTUs might represent multiple *Gangesia* specimens sequenced bearing an identical lsrDNA sequence. Rooted phylogram. Total nucleotide differences between individual isolates are summarized in the boxed table.

**Figure 11:**
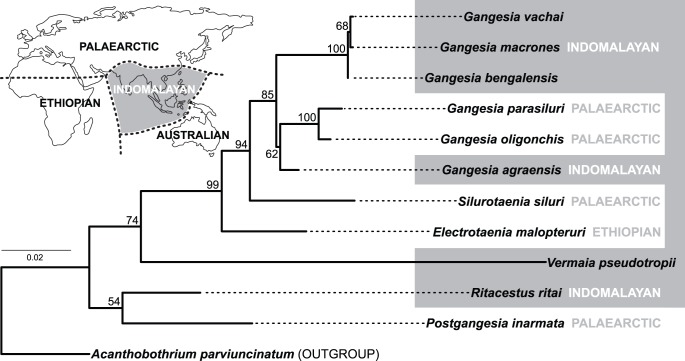
Phylogenetic interrelationships of the subfamily Gangesiinae based on lsrDNA data. *Gangesia* species with multiple specimens of unique lsrDNA sequence (*G*. *agraensis*, *G*. *oligonchis*, *G*. *vachai*) are depicted as single OTUs for clarity.

In addition, *S. vachai* forms a strongly supported clade with *Gangesia macrones* and *G. bengalensis* in molecular analyses, whereas *S. siluri* is a sister taxon of the clade formed by all species of *Gangesia*, including *S. vachai* (Figs. 10, 11). Therefore, molecular data support transfer of *S. vachai* to *Gangesia*, which well corresponds to its distribution, i.e. the Indomalayan Region (*S. siluri* occurs in the European part of the Palaearctic region).

*Gangesia vachai* is morphologically well differentiated from other *Gangesia* spp. by the presence of 4–6 irregular rows of rostellar hooks. Otherwise, it possesses similar proglottides and a small scolex like *G*. *macrones*, but can be easily differentiated by having a comparatively longer ovary (occupying about 1/3 of mature proglottid length) than the latter species (see Table 4).

The present authors found only two intact worms, but from two different hosts and localities, one from Durgapur, Bangladesh (field no. BAN 186) and another from Phulbari dam, Siliguri, India (field no. IND 906). They slightly differ in some morphological details (shape of the scolex, arrangement of hooks and distribution of hooklets of suckers; see Fig. 8A,D) and their lsrDNA sequences differed in 3 bp (0.2%), which is considered to represent intraspecific variability.

### Taxa of Uncertain Status

The systematic position and validity of three species of *Gangesia* and *Silurotaenia* described from India could not be confirmed. Their original descriptions were not available despite numerous requests to the editorial offices of relevant journals, the authors of the papers, and exhaustive search in internet databases and major libraries. Until these descriptions are available (type material is not known to have been deposited), the validity of each of the following species remains doubtful: (1) *Gangesia hanumanthai* Seth and Kapoor, 1982; (2) *G*. *chauhanii* Mathur and Srivastav, 2000; and (3) *Silurotaenia godavari* Wankhede and Jadhav, 2002.

Chavan [64] proposed two *Silurotaenia* species, namely *S*. *makniensis* and *S*. *gangakhedensis* from *W*. *attu*, in his unpublished PhD thesis and, thus, both of them are invalid according to the International Code of Zoological Nomenclature [72].

Wankhede [35] listed the species *Gangesia godavari* Kadam et al. 1983, but did not provide citation of the original description, which was unavailable to the present authors. In contrast, Bhure et al. [40] listed Jadhav, Shinde and Kadam (1983) as authorities of this species, but this paper [19] does not include any mention of *G*. *godavari*. However, the species is indistinguishable from *G. agraensis* as obvious from its brief description and figures (Figs. 2H and 3H) provided by Bhure et al. [40]. Therefore, *G. godavari* is considered to be a synonym of *G. agraensis*.

### Phylogenetic Relationships

Twenty-three 1556–1644 bp long lsrDNA sequences of *Gangesia* have been characterized within the scope of this study, including five samples of *Gangesia* spp. from other than the Indomalayan zoogeographical region. In addition, species of all remaining genera of the subfamily Gangesiinae (*Electrotaenia* Nybelin, 1942; *Postgangesia* Akhmerov, 1969; *Ritacestus* de Chambrier, Scholz, Ash and Kar, 2011; *Vermaia* Nybelin, 1942) have been sequenced and their sequences combined with those available in GenBank. Whereas no intraspecific variability was observed within *G. bengalensis* (n = 4; all samples from India) and *G. macrones* (n = 4; Bangladesh and India), sampled specimens of *G. agraensis* (n = 8; Cambodia and India) and *G. vachai* (n = 2; Bangladesh and India) differed in 1–5 and 3 bp, yielding 3 and 2 unique lsrDNA sequences per given species, respectively (see inset table in Fig. 10). Specimens of *G. oligonchis* from Russia (n = 5) were identical.

ML analyses of the *Gangesia* specimens studied (Fig. 10) confirmed the monophyletic status of each of the four redescribed *Gangesia* species from Indomalayan region. However, the observed genetic distances within individual species were not consistent (see inset table in Fig. 10). The morphologically noticeably distinct species *G. bengalensis*, *G. macrones* and *G. vachai* differed in a relatively low (4–6) number of nucleotides, but *G. agraensis* differed in 51–64 bp. The Palaearctic species (*G. oligonchis* and *G. parasiluri*) differed in 17 bp, whereas differences of these taxa from those of the Indomalayan region were markedly higher (36–51 bp from *G. agraensis* and 56–81 from the three remaining species).

The phylogenetic analysis of the representatives, usually type-species, of all genera of the subfamily Gangesiinae (Fig. 11) confirmed the monophyly of the genus *Gangesia*, represented here by six of nine currently valid species (see Discussion). Palaearctic *Silurotaenia siluri* appeared as a sister lineage to the *Gangesia* clade, *Electrotaenia malopteruri* then being a well-supported sister lineage of the two. Monophyly of the Indomalayan lineages of *Gangesia*, as detected on Fig. 10, was not revealed by the phylogenetic analysis involving all Gangesiinae. Here (Fig. 11), *G*. *agraensis* rather grouped with the Palaearctic *Gangesia* species, although without statistical support. Subsequent statistical topology test for a hypothesis constraining *G*. *agraensis* as a sister lineage to the rest of Indomalayan *Gangesia* species did not reject this scenario since the *p* value of the AU test reached a non-significant value of 0.423 (rejection at *p*<0.05).

## Discussion

The present study has revealed that the number of nominal species of proteocephalidean cestodes described as members of *Gangesia* and *Silurotaenia* from freshwater fishes in the Indomalayan region conspicuously exceeds that of actually valid taxa, similarly as in cestodes of the order Caryophyllidea (see [54], [73]). Instead of 48 spp. in two genera, only four species of *Gangesia* are considered to be valid. As many as 41 species are either invalidated as synonyms of one of the three species described between 1913 and 1928 or represent *nomina nuda*. It is also concluded, and molecular data strongly support this assumption, that no species of *Silurotaenia* occurs in India and neighbouring countries. In fact, all nominal species of *Silurotaenia* from the Indian subcontinent are synonyms of some of the four species of *Gangesia*. The genus *Silurotaenia* thus seems to be limited in its distribution to the Palaearctic region, with one species occurring in Europe and another one in China ([3], [70]).

In the silurid catfish *Wallago attu*, which is one of the most valuable fishes sold on markets in India [74], only two species of *Gangesia* occur commonly, which was first noticed by Verma [2]. All the remaining species described from this catfish appear to be merely synonyms of *G. bengalensis* or *G. agraensis*. Another economically important catfish, *Sperata seenghala*, has also been reported to host a number of proteocephalidean species, all but one (*G. macrones*) placed in *Silurotaenia*. However, it seems that the only valid species specific of *S. seenghala* is the species described by Woodland [1] and all seven species of *Silurotaenia* are its synonyms. The fourth species, *Gangesia vachai*, has been found in catfishes of as many as three families, but more data are necessary to clarify the host specificity and distribution of this apparently uncommon species. As a result of the present revision, a comparative table with differential characters of the four valid species parasitizing catfishes in the Indomalayan region is provided to facilitate their identification (Table 4).

It is unfortunate that all descriptions published after 1928 were inadequate, with many errors and were based on decomposed specimens with detached hooks and hooklets, and/or on deformed (contracted) tapeworms, the morphology of which did not reflect natural state. In addition, authors of these descriptions almost completely ignored previously published data, in particular a key paper by Verma [2], who first clarified the confused taxonomic situation of *Gangesia* tapeworms parasitic in *W. attu*.

Similarly as in other groups of cestodes parasitic in freshwater fishes in the Indian subcontinent [54] [73], there is indirect evidence that no type specimens of any species of *Gangesia* described during the last seven decades have been deposited in any collection. This is apparent violation of the basic rules of the International Code of Zoological Nomenclature [70] and editors of all journals declared to be scientific should avoid this deplorable practice.

*Gangesia* tapeworms have also been recorded from non-siluriform fishes, such as cyprinids (*Cirrhinus*, *Labeo*, *Puntius*) and the mastacembelid *Mastacembelus armatus*. However, no vouchers of any of these remarkable findings have been deposited, which makes these records doubtful. For this reason, it is impossible to assess reliably the actual role of these fish hosts in the transmission of *Gangesia* tapeworms, which have otherwise been found exclusively in catfishes ([3], [6], [75]). Misidentification of fish hosts (cyprinids and zig-zag eel versus siluriforms) is impossible but mislabelling of samples cannot be excluded.

Records of three *Gangesia* tapeworms, *G*. *batrachusi*, *G*. *clariusae* and *G*. *jayakwadensis*, which are newly synonymized with *G. agraensis* (first two) and *G*. *bengalensis* (last one), from the walking catfish, *Clarias batrachus*, are unreliable (no reference specimens exist). Mislabelling of the samples from *W. attu* seems to be the most probable explanation. It is worth mentioning that these three doubtful records were published by researchers from the laboratory, from which several tens [sic!] new species and several new genera of caryophyllidean tapeworms have been described from *C. batrachus*, all of them being in fact invalid (see [54]). The present authors dissected 235 *C. batrachus* and 105 specimens of other *Clarias* species from Bangladesh, Cambodia, India, Indonesia and Vietnam, but proteocephalidean tapeworms have never been found.

Future surveys, especially those in the Indian subcontinent, should focus on these atypical hosts, but adequate methods should be applied, including correct labelling of samples and their deposition in internationally accessible collections. The present authors have examined 363 specimens of six species of *Puntius*, 112 specimens of *Mastacembelus armatus* and 18 specimens of *Labeo* spp. from Bangladesh, India (Assam, Maharashtra and West Bengal) and Vietnam, but did not find any proteocephalideans.

Molecular lsrDNA data support the conclusions on the species composition of *Gangesia* tapeworms in the Indomalayan region based on morphological revision of type specimens and new material. Each of the four Indomalayan *Gangesia* species represents a monophyletic lineage with *G*. *bengalensis*, *G*. *macrones* and *G*. *vachai* forming a strongly supported clade (with low interspecific differences) apart from *G*. *agraensis* that most probably forms their sister lineage. The other strongly supported clade consists of two Palaearctic species, *G. oligonchis* from Russian Far East and *G*. *parasiluri* from Japan.

*Gangesia*, here represented by a majority of its species (six of nine), is a monophyletic assemblage, with European *Silurotaenia siluri* representing its sister taxon. Another strongly supported node found on the phylogenetic tree of the Gangesiinae represents *Electrotaenia* as a sister lineage to the clade composed of *Silurotaenia* and *Gangesia*. The phylogenetic relations of the remaining Gangesiinae, i.e. *Postgangesia*, *Ritacestus* and *Vermaia*, should remain considered as unclearly resolved.

*Gangesia* was one of the most species-rich genera of proteocephalidean cestodes with as many as 43 nominal taxa. However, the present study has demonstrated that the number of valid species is in fact much lower because as many as 32 species are invalidated. Based on the present revision, the genus consists only of *G*. *agraensis* Verma, 1928; *G*. *bengalensis* (Southwell, 1918); *G*. *macrones* Woodland, 1924; *G*. *margolisi* Shimazu, 1994; *G*. *oligonchis* Roitman and Freze, 1964; *G*. *parasiluri* Yamaguti, 1934; *G*. *polyonchis* Roitman and Freze, 1964; *G*. *pseudobagrae* Chen, 1962; and *G*. *vachai* (Gupta and Parmar, 1988). Validity of *G*. *spasskajae* Demshin, 1987, the type specimens of which are deposited in the Institute of Biology and Soil Science in Vladivostok, Russia, is questionable and requires confirmation.

The present study has also revealed that all Indian species of *Silurotaenia* are invalid, having been synonymized with some of the four species of *Gangesia*. The former genus thus seems to be restricted in its distribution to the Palaearctic region. *Gangesia vachai*, originally assigned to *Silurotaenia*, actually resembles in its morphology *S. siluri*, the type species of the genus, but this resemblance is apparently a result of convergence as evidenced by molecular data (Fig. 11). To better circumscribe individual genera of the Gangesiinae, a simple key based on morphological characteristics is provided.

### Key to the Genera of the Subfamily Gangesiinae Mola, 1929

1a. Rostellum-like organ with hooks .…………………………. 3
1b. Rostellum-like organ without hooks ………………………. 2
2a. Common genital atrium present; apical depression present on the top of the scolex; vagina always anterior to the cirrus-sac; type 2 uterine development (according to de Chambrier et al. 2004 [55]). In Bagridae (*Rita rita*). India ……………….…….………… *Ritacestus* de Chambrier, Scholz, Ash and Kar, 2011
2b. Common genital atrium absent, male and female genital pores open separately; apical depression on the top of the scolex absent; vagina usually posterior to the cirrus-sac; type 1 uterine development (according to de Chambrier et al. 2004). In Siluridae. Iraq, Russia ……….…………………………. *…….……….……….…….….….Postgangesia* Akhmerov, 1969
3a. Lateral band of vitelline follicles long, occupies almost total length of proglottides; proglottides anapolytic …………………….…. 4
3b. Lateral band of vitelline follicles short, limited only to the preovarian region, posterior to the genital pore; proglottides apolytic. In Schilbeidae (*Clupisoma garua*), India …………….…………………………………………. *Vermaia* Nybelin, 1942
4a. Testes form one field; rostellum-like organ spherical …………….……………………………………….….….… 5
4b. Testes form two fields; rostellum-like organ disc-shaped (flattened). In Malapteruridae (*Malapterurus electricus*). Africa ……………………………….…… *Electrotaenia* Nybelin, 1942
5a. Anterior rim of suckers covered with hooklets; rostellum-like organ larger than or as large as suckers; ventral osmoregulatory canals median (internal) to vitelline follicles; strong retractor muscles form a wide band. In Siluridae, Bagridae and Schilbeidae. Bangladesh, Cambodia, China, India, Japan, Pakistan, Russia, Sri Lanka ………. *Gangesia* Woodland, 1924
5b. Anterior rim of suckers without hooklets (covered with microtriches only); rostellum-like organ markedly smaller than suckers; ventral osmoregulatory canal lateral (external) to vitelline follicles; retractor muscles only on lateral sides of rostellum-like organ. In Siluridae (*Silurus glanis*, *S*. *soldatovi*?). Europe, China? …………….….…. *Silurotaenia* Nybelin, 1942
